# Exploring the molecular mechanism of Coptis-cinnamon in combating gastric cancer via the MAPK Pathway based on network pharmacology

**DOI:** 10.3389/fonc.2026.1824009

**Published:** 2026-05-14

**Authors:** Zhao-zhao Wang, Ya-hong Li, Ling Yuan, Shu-min Jia, Peng Yang, Wen-jing Liu, Yi Nan

**Affiliations:** 1 Key Laboratory of Dryness Syndrome in Chinese Medicine, Ministry of Education, Ningxia Medical University, Yinchuan, Ningxia, China; 2 College of Traditional Chinese Medicine, Ningxia Medical University, Yinchuan, Ningxia, China; 3 College of Pharmacy, Ningxia Medical University, Yinchuan, Ningxia, China

**Keywords:** coptis-cinnamon, gastric cancer, *in vitro* validation, MAPK pathway, network pharmacology

## Abstract

**Background:**

Gastric cancer (GC) ranks as the fifth most common malignancy worldwide. Current treatments are limited by side effects and drug resistance, highlighting the need for novel therapies. Traditional Chinese medicine (TCM) pair Coptis-Cinnamon (HL-RG) shows promise against GC, but its mechanism remains unclear.

**Methods:**

Active components and targets of HL-RG were obtained from TCMSP, and GC-related genes from GEO and GeneCards. Intersection targets were analyzed via STRING for PPI network construction. GO and KEGG enrichment analyses were performed using DAVID to identify key pathways and hub genes. Clinical relevance, mutations, immune infiltration, and drug sensitivity were analyzed. Molecular docking validated interactions between core components and hub genes. *In vitro* experiments (CCK-8, flow cytometry, Transwell, qRT-PCR, Western blot) were conducted in AGS and HGC-27 cells to verify anti-GC effects. To verify the causal relationship, we conducted rescue experiments using the MEK inhibitor U0166 and the MAPK agonist EGF.

**Results:**

Sixteen active components and 499 targets of HL-RG were identified, with 55 common targets screened from 3,194 GC-related DEGs. Enrichment analyses revealed involvement in inflammatory responses and the MAPK pathway, identifying PDGFRB, EGFR, MMP2, MMP9, and KIT as hub genes. Molecular docking showed binding affinities between core components (berberine, berberrubine) and hub genes. *In vitro* experiments confirmed that HL-RG inhibits GC cell proliferation, induces apoptosis, and suppresses migration and invasion via MAPK pathway regulation. Rescue experiments demonstrated that U0126 phenocopied HL−RG effects, while EGF significantly reversed HL−RG−induced functional changes and p−ERK reduction, confirming that HL−RG acts through the MAPK/ERK pathway.

**Conclusions:**

HL-RG exerts anti-GC effects by targeting the MAPK pathway and its hub genes, providing a scientific basis for its clinical application.

## Graphical Abstract

**Figure:**
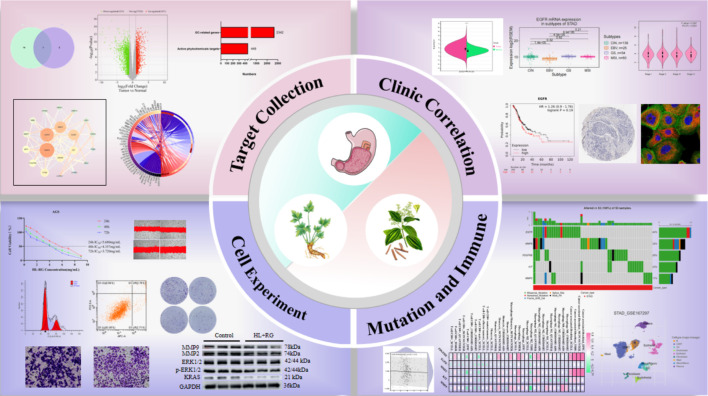

## 1 Introduction

GC is a prevalent gastrointestinal malignancy and a leading cause of cancer-related morbidity and mortality worldwide, ranking as the fifth most common cancer and the fifth leading cause of cancer-related deaths. Its development is influenced by factors such as family history, diet, alcohol consumption, and Helicobacter pylori infection (1). Current treatment modalities include chemotherapy, radiation therapy, targeted therapy, and immunotherapy. However, patient survival and prognosis remain suboptimal due to delayed diagnosis, drug resistance, and side effects such as intolerance to radiotherapy and chemotherapy. Accumulating evidence suggests that TCM offers advantages in the treatment of GC through its multi-target, multi-pathway, and multi-effect characteristics, making it an integral part of the comprehensive treatment system for this disease. Further investigation into the clinical efficacy of TCM, comprehensive exploration of its underlying molecular mechanisms, and identification of novel therapeutic targets are of great significance for improving the clinical management of GC (2).

Coptis (HL), first documented in the *Shennong Ben Cao Jing*, is a classical herbal medicine traditionally used to treat gastrointestinal disorders. It is regarded as a superior herb (nourishing and nontoxic) and is a key component in many TCM formulas, such as Gegen Qinlian Decoction and Coptis Jiedu Decoction. According to TCM theory, HL is commonly used to clear dampness, drain heat, reduce fire, and detoxify (3). As dampness-heat, fire, and toxins are considered external pathogenic factors in TCM, the therapeutic effects of HL are often attributed to its anti-inflammatory and antibacterial activities (4).

Although the pathological concept of “tumor” is a modern medical term, descriptions resembling cancer symptoms can be found in ancient Chinese medical records. For instance, the *Zhong Zang Jing* describes conditions such as “carbuncles, sores, and swellings,” which are believed to arise from the accumulation of various pathogens, including heat and dampness, in the body. Based on this understanding, the traditional use of HL for clearing heat and dampness suggests its potential application in treating cancer. This ethnopharmacological link is further supported by its clinical use in alleviating diarrhea, vomiting, fever, and bleeding—symptoms commonly observed in cancer patients. The consistency between the pathogenesis described in TCM and the symptomatic use of HL reinforces the rationale for investigating its antitumor activity (5).

Recent studies have demonstrated significant progress in understanding the anticancer effects, mechanisms, and clinical applications of HL (6). It has been shown to effectively inhibit the proliferation, invasion, and migration of GC cells. Its active constituents regulate the cell cycle, cell differentiation, and metastasis while enhancing anti-inflammatory and immune functions, thereby exerting antitumor effects against GC and its complications (7). To date, over 100 chemical constituents have been isolated from HL, including flavonoids, alkaloids, lignans, and acidic components. Among these, alkaloids are the primary pharmacologically active components. Berberine, the most representative and abundant alkaloid in HL, exhibits antibacterial, anti-inflammatory, and antitumor properties (8) and has demonstrated potent activity against various human malignancies, including GC (9).

The dried bark of *Cinnamomum cassia (L.) J.Presl*, known as Cinnamomi Cortex (RG), is derived from a plant belonging to the Lauraceae family and is used both as a food spice and a medicinal herb. RG has been cultivated and utilized worldwide for centuries, with its earliest records found in the *Shennong Ben Cao Jing*, where it was regarded as a Chinese herbal medicine of high medicinal value.

According to the *Chinese Pharmacopoeia*, RG is warm in nature, pungent and sweet in taste, and acts on the spleen, kidney, heart, and liver meridians. Its functions include tonifying fire to assist yang, guiding fire back to its origin, dispelling cold to alleviate pain, and warming the meridians. Clinically, RG is often used to treat arthritis, dizziness, vomiting, fever, diarrhea, abdominal pain, heart disease, prostatitis, dysmenorrhea, and amenorrhea (10). Phytochemical studies have shown that its main active components include essential oils (11), polyphenols (12), diterpenoids (13), flavonoids (14), polysaccharides (15), and other constituents (16). Pharmacological investigations have demonstrated that RG possesses a diverse array of bioactive compounds with anti-inflammatory, antibacterial, antioxidant, and antitumor activities^[^ (17–19).

For instance, researchers have reported that cinnamaldehyde can induce apoptosis in colon cancer cells by inhibiting the PI3K/Akt signaling pathway. Additionally, cinnamaldehyde upregulates the expression of E-cadherin while downregulating MMP2 and MMP9 (20). *In vitro* studies have also shown that cinnamaldehyde slows the growth of human lung cancer cell lines in a dose-dependent manner (21). Furthermore, cinnamaldehyde induces apoptosis in cancer cells by increasing intracellular ROS levels (22). Other studies have revealed that RG bark extract can inhibit the proliferation of breast cancer cells and induce apoptosis *in vitro (*23). Additionally, compounds developed based on the structure of natural products such as cinnamic acid and piperidinic acid have demonstrated moderate anti-proliferative effects on the HCT116 cell line, which were further confirmed in HT29, LoVo, and SW480 cells (24).

In addition to berberine, other natural products have recently been reported to exert anti−gastric cancer effects through distinct mechanisms. Morin, a flavone extracted from Prunella vulgaris, was shown to inhibit gastric cancer cell growth by blocking the ubiquitination−based degradation of the pro−apoptotic protein BAD, thereby inducing intrinsic apoptosis (25). Cycloastragenol, a tetracyclic triterpene saponin from Astragali Radix, has also been reported to possess antitumor activity in GC (26). These findings further support the therapeutic potential of phytochemicals against this malignancy.

Although previous studies have demonstrated that berberine or cinnamaldehyde alone can inhibit GC cells via MAPK−related pathways, these investigations focused on single compounds, overlooking the synergistic effects of the herbal pair in clinical practice. HL−RG is a classic Chinese medicinal pair known for its mutually reinforcing and counteracting compatibility. However, its anti−GC effect has never been systematically studied as an integrated formula. Investigating HL−RG as a whole may reveal multi−target network regulation and enhanced bioavailability between components, which cannot be achieved by any single ingredient.

In 2003, Professor Li Shao (27) of Tsinghua University proposed a “Bioinformatics Research Strategy for Complex Diseases” to analyze the “network target-system regulation” mechanism of drugs. This approach posits that drug components may act on network targets associated with specific phenotypes, thereby affecting the static key structure or homeostasis of these targets and systematically interfering with the phenotype at the cellular or organismal level. Subsequently, the concept of “network targets” was further developed by Professor Hopkins (28), and in 2021, the first “Network Pharmacological Evaluation Method Guide” was published (29). The integration of active ingredient targets from Chinese herbal medicines with disease-related biological networks has opened new horizons for exploring the application of herbal compounds in disease treatment.

In this study, we utilized publicly available datasets and combined bioinformatics analysis with network pharmacology to identify anti-tumor targets and signaling pathways associated with HL-RG. We thoroughly examined the clinical relevance, gene mutations, and immune infiltration characteristics of these targets. To further evaluate HL-RG’s inhibitory effect on GC, we conducted cell cycle, apoptosis, and colony formation assays using GC cell lines AGS and HGC-27 to assess cell proliferation capacity. Additionally, wound healing, migration, and invasion assays were performed to evaluate metastatic potential. Results demonstrated that HL-RG significantly suppressed GC cell proliferation and metastatic potential. *In vitro* studies further revealed that HL-RG may exert antitumor effects by regulating the RAS/RAF/MEK/ERK signaling pathway. To further establish the causal role of the MAPK pathway in HL-RG mediated anti-GC effects, we performed rescue experiments using the MEK inhibitor U0126 and the pathway agonist EGF. The workflow of this study is illustrated in Graphical Abstract.

## 2 Materials and methods

### 2.1 Acquisition of drug and disease targets

#### 2.1.1 Screening of active ingredients and targets of HL-RG

The active ingredients of HL-RG were obtained from the Traditional Chinese Medicine Systems Pharmacology (TCMSP) database (https://www.tcmsp-e.com/). Ingredients meeting the criteria of oral bioavailability (OB) ≥ 30% and drug-likeness (DL) ≥ 0.1 were selected as the candidate active compounds. Corresponding protein targets of these ingredients were also retrieved from TCMSP. The targets common to both HL-RG were identified as the potential synergistic targets of HL-RG. A Venn diagram illustrating this intersection was generated using an online bioinformatics platform (https://www.bioinformatics.com.cn/). Finally, a network diagram visualizing the relationships among HL-RG, their active ingredients, and the common targets was constructed using Cytoscape software (version 3.10.0).

#### 2.1.2 Screening of GC targets

First, the GEO database (https://www.ncbi.nlm.nih.gov/geo/) was searched using “gastric carcinoma” and “GC” as keywords to obtain GC-related gene expression datasets. Genes with P < 0.05 and |logFC| ≥ 1 were defined as differentially expressed genes (DEGs). Genes with logFC ≥ 1 were considered upregulated, and those with logFC ≤ -1 were considered downregulated. GraphPad Prism 10 software was then used to generate a volcano plot to visualize the expression levels of all genes. Additionally, using “stomach carcinoma” as the keyword, GC-related targets were obtained from the DisGeNET database and the GeneCards website, and the intersections were combined.

#### 2.1.3 Identification of core targets

The numbers of HL-RG drug targets and GSE65801-derived DEGs were summarized using a bar graph generated with GraphPad Prism 10. To identify potential core targets, we first intersected the HL-RG targets with the upregulated and downregulated DEGs from the GSE65801 dataset using the BioLadder online platform (https://www.bioladder.cn). Subsequently, a comprehensive intersection analysis was performed using the jvenn tool (http://jvenn.toulouse.inra.fr/app/index.html) to find common targets among three sets: (1) HL-RG targets, (2) GSE65801 DEGs, and (3) GC-associated genes from DisGeNET and GeneCards.

The expression levels of these common intersection targets in GC versus normal tissues were visualized as a heatmap using BioLadder, with genes ranked by their log_2_FC values. The top 10 upregulated and top 10 downregulated genes were listed. Finally, to assess the global expression differences between the GC and normal groups, principal component analysis (PCA) was performed on these intersection targets using the BioLadder platform.

### 2.2 Screening and enrichment analysis of hub genes

#### 2.2.1 Construction of a PPI protein interaction network diagram

Using the STRING database, we constructed an interaction network diagram of the targets involved in the treatment of GC by HL-RG. A topological network diagram was constructed using Cytoscape 3.10.0 software with the CentiScaPe 2.2 plugin to calculate network topology parameters including betweenness centrality, closeness centrality, and degree. A degree ≥ 7.259259 was used to determine the final key targets, and the degree values of these key targets were displayed in a bar chart.

#### 2.2.2 Correlation analysis of core targets

To elucidate the interrelationships among the core targets of HL-RG in GC treatment, we performed correlation analysis. The expression correlation of these core targets in GC samples was analyzed using the GEPIA2 database (http://gepia2.cancer-pku.cn/#index). A heatmap visualizing the correlation matrix among the targets was generated using the ChiPlot online platform (https://www.chiplot.online/).

#### 2.2.3 GO and KEGG enrichment analysis

To explore the biological functions of the core targets, we performed Gene Ontology (GO) functional annotation and Kyoto Encyclopedia of Genes and Genomes (KEGG) pathway enrichment analysis. The core targets were submitted to the DAVID database (https://davidbioinformatics.nih.gov/) for enrichment analysis, covering biological process (BP), cellular component (CC), and molecular function (MF) terms. The enrichment results were visualized using the Sangerbox platform (http://sangerbox.com/), including a circle diagram for GO terms and a Sankey bubble diagram for KEGG pathways.

### 2.2.4 Pathway and GSEA enrichment analysis

The pathway with the highest number of enriched targets was selected from KEGG Mapper (https://www.genome.jp/kegg/mapper/) for further analysis. GSEA enrichment analysis was subsequently performed on all GC-associated targets. Using the CAMOIP website (http://www.zjyy-oncology.com:20002/), we analyzed the associations of each core target with GC and the MAPK signaling pathway.

### 2.3 Relationships between hub genes and clinical outcomes

#### 2.3.1 Correlation of hub genes with clinical features

To analyze the relationships between the hub genes and the occurrence, type, and prognosis of GC, we performed a series of bioinformatics analyses. First, nonparametric tests were performed on samples from the TCGA and GTEx databases via the Sangerbox platform to evaluate the correlation between the mRNA expression levels of the hub genes and GC. Subsequently, the association between copy number variations of the hub genes and GC was examined using the GEPIA2 website. Next, differential expression of the hub genes at the protein level was verified using the CPTAC module of the UALCAN website. The GSCA website (https://guolab.wchscu.cn/GSCA/#/) was then used to evaluate the relationship between hub gene expression and GC subtypes. Finally, the correlation between the clinical stage of GC and hub gene expression was analyzed using the GEPIA2 website.

#### 2.3.2 Pathological validation and prognostic analysis of hub genes

To validate the expression of the hub genes at the protein level, we utilized the Human Protein Atlas (HPA) database (https://www.proteinatlas.org/). Immunohistochemistry (IHC) images of GC and normal gastric glandular tissues were obtained to analyze the protein expression patterns of the core genes. Additionally, immunofluorescence images available in the HPA database were examined to study the subcellular localization of these hub genes in tumor tissues.

#### 2.3.3 Prognostic analysis of hub genes

To further explore the correlation between hub gene expression and the prognosis of GC patients, Kaplan–Meier survival analysis was performed using the KM-plotter website (http://kmplot.com/analysis/). The overall survival or relapse-free survival of patients stratified by high and low expression of each hub gene was compared, and the results were visualized as Kaplan–Meier survival curves.

### 2.4 Correlations between hub gene mutations and GC

### 2.4.1 Hub gene mutation analysis

To investigate the correlation between GC and hub gene mutations, we performed a detailed analysis using the GSCA database. Single nucleotide variants (SNVs) were classified as deleterious or non-deleterious mutations. First, we analyzed the mutation sites and types of SNVs in the hub genes, and the frequency of deleterious mutations was visualized as a percentage heatmap. To further explore the impact of copy number variations (CNVs), mutation data were processed using the GISTIC 2.0 algorithm, and the results were presented as bubble plots.

Tumor tissues typically consist of both driver mutation genes and passenger mutation genes. Driver genes confer a selective growth advantage to tumor cells during the development and progression of GC and thus play a crucial role in tumorigenesis. Using the CAMOIP database, we identified the top 20 driver mutation genes closely associated with the hub genes. These included oncogenes, tumor suppressor genes, and genes of unknown classification.

#### 2.4.2 Hub gene methylation analysis

The UALCAN website (https://ualcan.path.uab.edu/analysis.html) was used to investigate the relationship between hub gene methylation and GC. The results were presented as boxplots generated from TCGA methylation data. The TIDE website (http://tide.dfci.harvard.edu/) was used to analyze the correlation between hub gene methylation and cytotoxic T lymphocyte (CTL) infiltration, as well as the survival differences between hypomethylation and hypermethylation groups in GC.

#### 2.4.3 Mutation repair system

Although gene mutations affect the occurrence and progression of tumors, damaged genes are regulated by existing repair systems *in vivo*, such as homologous recombination repair (HRR) and mismatch repair (MMR). GEPIA2 was used to calculate the correlations between the hub genes and repair genes, and the ChiPlot website (https://www.chiplot.online/) was used to draw a correlation heatmap.

### 2.5 Immune correlation of hub genes

#### 2.5.1 Immune infiltration and immune checkpoints

The CIBERSORTx website (https://cibersortx.stanford.edu/) was used to estimate the abundances of 22 immune cell types in mixed-cell subtypes. Data obtained from the GEO database were input into the CIBERSORT online platform for computational analysis, and the results were visualized as box plots. Pancancer analysis was then performed using the Sangerbox database to construct scatter plots for stromal score, immune score, and ESTIMATE score evaluation related to immune cells. Additionally, a heatmap of the relationships between GC-related core genes and immune checkpoints was generated.

#### 2.5.2 Single-cell sequencing analysis

To investigate the expression of core targets at the single-cell level, we utilized the TISCH2 database (http://tisch.comp-genomics.org/home/). Single-cell RNA sequencing (scRNA-seq) datasets containing GC samples were selected. Annotated single-cell profiles of the core targets in the GC tumor microenvironment were obtained and visualized using parameters available on the TISCH2 platform.

#### 2.5.3 Correlation between immune cells and GC

The TIMER 2.0 database (http://timer.cistrome.org/) was used to analyze the correlations between the hub genes and the infiltration levels of various immune cells and stromal cells in GC. Specifically, we examined the associations with macrophages, CD8+ T cells, cancer-associated fibroblasts (CAFs), and endothelial cells. The resulting correlation matrix was visualized as a heatmap generated using the ChiPlot platform (https://www.chiplot.online/).

### 2.6 Drug sensitivity analysis

First, the TISIDB (http://cis.hku.hk/TISIDB/index.php) website was used to analyze the correlations between the hub genes and immunotherapy response. Given that IFN-γ can promote tumor cell destruction and TGF-β can induce tumor cell apoptosis and inhibit tumor cell proliferation, the correlations between the hub genes and these two cytokines were calculated using the CAMOIP website. Additionally, the relationships between hub gene expression and drug sensitivity (agility) in anticancer drugs were downloaded from the GSCA database, and the results were visualized as bubble plots.

### 2.7 Molecular docking

The 2D structures of the active ingredients were obtained from the PubChem database (https://pubchem.ncbi.nlm.nih.gov/) and converted into 3D structures using ChemOffice software, then saved as mol2 files. Protein crystal structures of hub genes with high resolution were retrieved from the RCSB PDB database (http://www.rcsb.org/). These protein structures were preprocessed using PyMOL software to remove water molecules and phosphate groups, and then saved as PDB files. Molecular docking was performed using AutoDock Vina 1.5.6 software to explore protein–ligand interactions. Using AutoDock tools, the protein structures were prepared by adding hydrogen atoms and removing water molecules, while the small molecule ligands were prepared by adding hydrogen atoms and determining the number of rotatable bonds. The docking box coordinates were defined based on the original ligand binding sites. The optimal binding conformations were selected by comparing the docking scores (binding energies, kcal/mol).

Redocking validation: To validate the reliability of the docking protocol, the co-crystallized ligands of EGFR and MMP2 were extracted and re-docked into the same binding pocket using the same parameters. The root−mean−square deviation (RMSD) between the re-docked pose and the original crystal pose was calculated. An RMSD value < 2.0 Å was considered acceptable, confirming that the docking parameters can accurately reproduce the experimental binding modes. After the binding pockets were validated, the other active ingredients of HL-RG were docked into the same pockets, and the resulting docking scores were calculated.

The 2D interaction diagrams were generated using Discovery Studio 2019, and 3D visualizations were prepared with PyMOL software.

### 2.8 Experimental materials and methods

#### 2.8.1 Cell culture

The human gastric mucosal epithelial cell line GES-1 was cultured in DMEM. The GC cell lines AGS and HGC-27 were cultured in DME/F-12 and RPMI-1640 medium, respectively. All media were supplemented with 10% FBS and 1% penicillin–streptomycin. All cell lines were authenticated by short tandem repeat (STR) profiling. Cells were maintained in a humidified incubator at 37 °C with 5% CO_2_.

#### 2.8.2 HL-RG preparation

The method for dissolving HL-RG was previously described by our team (30). The formula granules of HL-RG were purchased from Yinchuan Hospital of Traditional Chinese Medicine and manufactured by Guangdong Yifang Pharmaceutical Co., Ltd. The granules were dissolved in 30 mL of ddH_2_O at a 1:1 ratio, vortexed, and then fully dissolved in warm water. The mixture was centrifuged at 12000 rpm for 10 min, and the supernatant was collected and filtered through a 0.22 μm filter. The resulting solution was aliquoted into 1.5 mL centrifuge tubes and stored at -80 °C.

### 2.9 Cell proliferation and apoptosis assays

#### 2.9.1 Cell viability measured by CCK-8 assay

AGS and HGC-27 GC cells and GES-1 normal gastric epithelial cells in logarithmic growth phase were seeded into 96-well plates at a density of 4 × 10^4^ cells/mL and cultured at 37 °C for 24 h. The cells were then treated with HL-RG at concentrations ranging from 0 to 7 mg/mL for 24, 48, or 72 h. For positive control, cells were treated with 5-fluorouracil (5-FU) at concentrations ranging from 0 to 50 μg/mL for 24, 48, or 72 h. Subsequently, 10 μL of CCK-8 reagent and 90 μL of fresh medium were added to each well, and the plates were incubated at 37 °C for an additional 2 h. Optical density (OD) at 450 nm was measured using a microplate reader. Cell viability was calculated as: (OD_treated_ – OD_blank_)/(OD_control_ – OD_blank_) × 100%. Each experiment was performed in triplicate to ensure accuracy.

#### 2.9.2 Apoptosis and cell cycle analysis by flow cytometry

AGS and HGC-27 cells in logarithmic growth phase were seeded into 6-well plates and treated with different concentrations of HL-RG (AGS: 3, 5, and 7 mg/mL; HGC-27: 3, 5, and 7 mg/mL) for 24 h. For apoptosis detection, cells were collected and stained using an Annexin V-FITC/PI apoptosis kit according to the manufacturer’s instructions. For cell cycle analysis, cells were starved in serum-free medium for 6–8 h to achieve synchronization prior to HL-RG treatment. After treatment, cells were collected, fixed in 70% ethanol overnight at 4 °C, and stained with a cell cycle detection kit. Flow cytometry was then performed to analyze cell cycle distribution. All experiments were performed in triplicate.

#### 2.9.3 Colony formation assay

AGS and HGC-27 cells in logarithmic growth phase were seeded into 6-well plates at a density of 800 cells per well. After 48 h of incubation, the cells were treated with HL-RG. The culture was terminated when the number of cell colonies in each well reached ≥ 50 under microscopic observation. The cells were washed three times with PBS, and colonies with a diameter > 1 mm were photographed and counted. The experiment was performed in triplicate.

### 2.10 Cell migration ability assay

#### 2.10.1 Wound healing assay

AGS and HGC-27 cells in logarithmic growth phase were seeded into 6-well plates. After 24 h of culture, when cells reached confluence, a linear wound was created by scratching the cell monolayer with a 10 µL pipette tip. The detached cells were removed by washing with PBS, and then HL-RG was added to the wells. The cells were cultured and images of the scratched areas were captured under a microscope at 0, 24, and 36 h. Cell migration ability was evaluated by calculating the wound closure rate. The experiment was performed in triplicate.

#### 2.10.2 Migration and invasion assays

Migration assay:AGS and HGC-27 cells in logarithmic growth phase were harvested and resuspended in serum-free medium at a density of 5 × 10^4^ cells/mL. A 100 µL aliquot of the cell suspension was seeded into the upper chamber of a Transwell insert (8 µm pore size). The lower chamber was filled with 800 µL of medium containing 30% FBS as a chemoattractant. After 24 h of incubation, the medium in the lower chamber was replaced with fresh medium containing HL-RG, and the cells were incubated for another 24 h. The cells on the upper surface of the membrane were then removed with a cotton swab. The migrated cells on the lower surface were fixed with 4% paraformaldehyde for 30 min, stained with 0.1% crystal violet for 30 min, washed with PBS, air-dried, and photographed under a microscope.

Invasion assay:For the invasion assay, Matrigel was diluted according to the manufacturer’s instructions and evenly spread onto the upper chamber of the Transwell insert. The insert was then placed at 4 °C for 4 h to allow the Matrigel to solidify. The subsequent steps were the same as those described for the migration assay. All experiments were performed in triplicate.The information of specific experimental materials and reagents is shown in **Table 1**.

**Table 1:** Experimental materials and reagents.

Materials and reagents	Manufacturer	Item number
RPMI-1640	Invitrogen	IN0010
DME/F-12	Cytiva Corporation of the United States	SH30023.01
PBS buffer	Cytiva Corporation of the United States	SH30256.01
Trypsin - EDTA	Beijing solarbio Company	T1320
Fetal bovine serum	GEMINI	900108
Matrigel	Biozellen Corporation of the United States	B-P-00002-10
4% polyformaldehyde fixing solution	Jinan century Tongda Chemical Co., LTD	30525–89-4
CCK-8 kit	KeyGEN BioTECH, China	KGA9305-500
Cell apoptosis detection kit	KeyGEN BioTECH, China	KGA106
Cell cycle test kit	KeyGEN BioTECH, China	KGA512
Trizol reagent	ThermoFisher Scientific of the United States	343,706
PrimeScript RT kit	Takara,Japan	RR047A
SYBR Premix Ex Taq TM II kit	Takara,Japan	RR820A
Whole Cell Lysis Assay	KeyGEN BioTECH, China	KGB5303-50
BCA Protein Quantitation Assay	KeyGEN BioTECH, China	KGB2101-50

### 2.11 qRT–PCR

TRIzol reagent was used to extract total RNA from AGS cells. Both the qPCR kits and the qRT–PCR kits were purchased from Takara Corporation. Using GAPDH as an internal reference, the amount of RNA was calculated via the 2^-ΔΔCT^ method. This step was repeated three times.The primer sequences used are detailed in **Table 2**.

**Table 2:** qRT-PCR primer sequence information.

Genes	Forward	Reverse
EGFR	TCGGCACGGTGTATAAGGGACTC	ACGGTGGAGGTGAGGCAGATG
MMP9	CTGGTCCTGGTGCTCCTGGTG	CTGCCTGTCGGTGAGATTGGTTC
MMP2	CACCTACACCAAGAACTTCCGTCTG	GTGCCAAGGTCAATGTCAGGAGAG
KIT	GGCGACGAGATTAGGCTGTTATG	CGGTGTTGGTGGCTTCTGC
PDGFRB	TTACCACATCCGCTCCATCCTG	ATTCACACTCTCCGTCACATTGC

### 2.12 Western blot

AGS cells in logarithmic growth phase were treated with HL-RG for 24 h. After treatment, the cell culture supernatant was collected, and total protein was extracted using RIPA lysis buffer supplemented with protease inhibitors. Protein concentration was determined using a BCA protein assay kit according to the manufacturer’s instructions. Equal amounts of protein were separated by SDS-PAGE and then transferred onto PVDF membranes. The membranes were blocked with rapid blocking solution for 15 min at room temperature, followed by incubation with primary antibodies overnight at 4 °C. After washing, the membranes were incubated with horseradish peroxidase (HRP)-conjugated secondary antibodies for 90 min at room temperature. Protein bands were visualized using enhanced chemiluminescence (ECL) reagent, and the signal intensity was quantified using ImageJ software. All experiments were performed in triplicate.The specific primary antibody information is shown in **Table 3**.

**Table 3:** Experimental antibody information.

Antibody	Antibody manufacturer	Article number
EGFR	Abmart	T55112F
KIT	Abways	CY5359
MMP2	Affinity	AF5330
MMP9	Affinity	AF5228
ERK1/2	Affinity	AF0155
p-ERK1/2	Affinity	AF1015
KRAS	Affinity	DF6324
PDGFRB	Wuhan Sanying	82943-1-RR

### 2.13 MAPK pathway rescue experiments

To verify whether the anti−gastric cancer effects of HL−RG are mediated by the MAPK signaling pathway, rescue experiments were performed using the MEK inhibitor U0126 and the pathway agonist EGF. AGS and HGC−27 cells were pretreated with U0126 (10 µM for AGS, 30 µM for HGC−27) for 1 hour, or with EGF (20 ng/mL for AGS, 40 ng/mL for HGC−27) for 30 minutes, followed by co−treatment with HL−RG for 24 or 48 hours as indicated. After treatment, cell proliferation was assessed by CCK−8 assay, cell cycle analysis (flow cytometry), apoptosis detection (Annexin V−FITC/PI staining), and colony formation assay. Cell migration and invasion were evaluated by wound healing and Transwell assays. The protein expression levels of p−ERK1/2 and total ERK1/2 were determined by Western blot. All experiments were performed in triplicate.

## 3 Results

### 3.1 Prediction and screening of targets of HL-RG and GC

**Figure 1:**
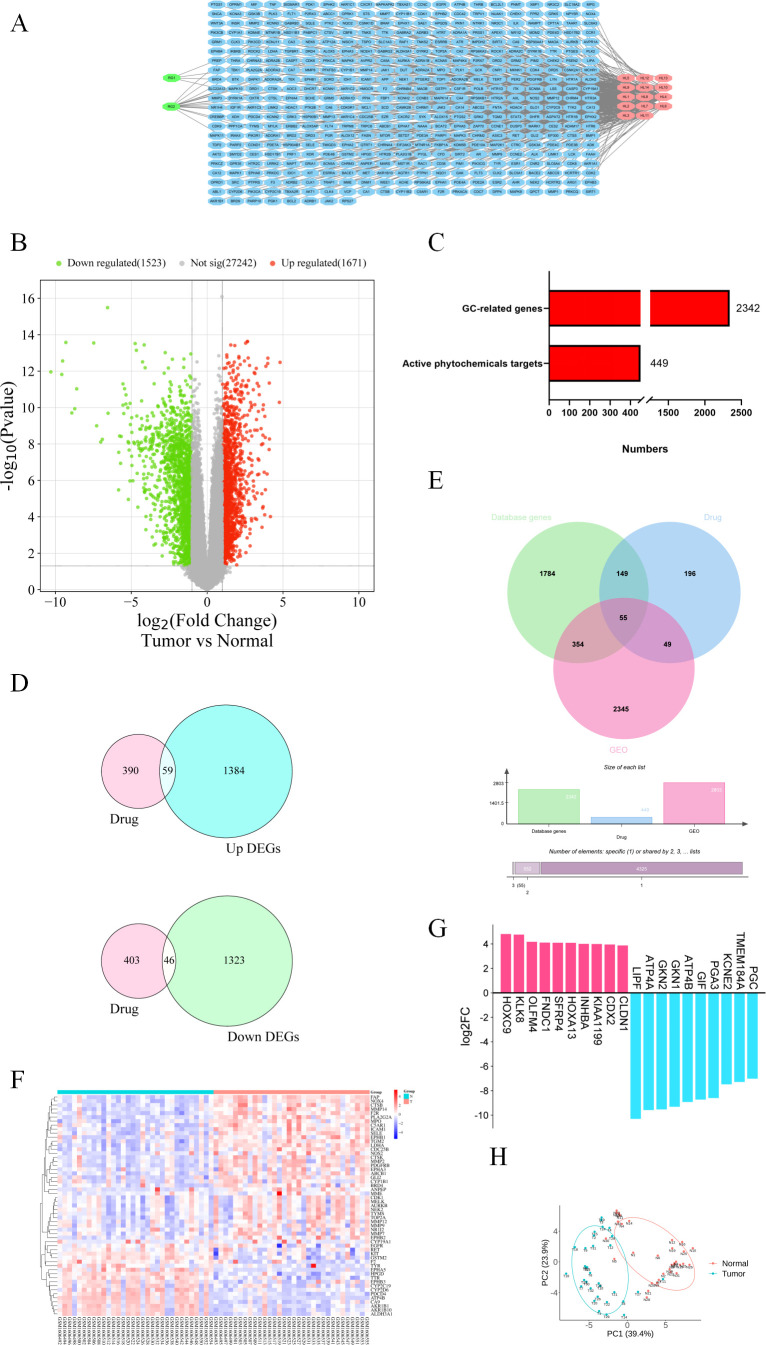
Targets of HL-RG active ingredients and differentially expressed genes in gastric cancer. **(A)** Network diagram of HL-RG active ingredient-targets. Green and red colors represent RG and HL, respectively, and blue color represents all the targets of active ingredients. **(B)** Volcano plot of differentially expressed genes of GSE65801. Where green represents down-regulated genes, red represents up-regulated genes, and gray indicates no difference or no significance. **(C)** All genes of gastric cancer and the number of targets of HL-RG active ingredients. **(D)** Intersection of up- and down-regulated genes of gastric cancer and targets of HL-RG. The pink color represents the targets of the compound, and the blue and green colors represent the up- and down-regulated genes, respectively. **(E)** Intersection plot of HL-RG and gastric cancer targets. Green and pink represent Database genes and GEO datasets, respectively, and blue represents HL-RG. **(F)** Heatmap of intersecting targets. Red and blue represent the tumor group and normal group, respectively. **(G)** LogFC values of the top 10 up- and down-regulated genes in the intersection targets, where red and blue represent up- and down-regulated genes, respectively. **(H)** PCoA plots of the samples of the intersecting targets. Red dots represent normal group samples and green dots represent tumor group samples.

First, we obtained a total of 16 active components from HL-RG from the TCMSP database. Detailed information on these active components is provided in **Supplementary Materials Table 1**. As shown in **Figure 1A**, the number of active components differed between the two drugs, with HL having 14 and RG having 2. Next, we constructed an active component–target network diagram for HL-RG using Cytoscape software, In this diagram, the blue nodes in the middle represent targets, while the green and red nodes represent the active components of HL-RG, respectively. Through this analysis, we identified a total of 499 targets that play key roles in the pharmacological effects of HL-RG.

We analyzed the dataset GSE65801 from the GEO database, which included a total of 64 samples (32 normal samples and 32 GC samples). As illustrated in **Figure 1B**, the GSE65801 dataset contains a total of 30,436 genes related to GC, with green indicating downregulated genes, red indicating upregulated genes, and gray indicating genes with no significant difference. Through this analysis, we identified a total of 3194 differentially expressed genes (DEGs). Additionally, a total of 3076 GC-related genes were obtained from the GeneCards and DisGeNET databases.

Finally, the total numbers of GC-related targets and drug-related targets are summarized in **Figure 1C**, with 2342 targets for GC and 449 targets for HL-RG.

### 3.2 Construction of the intersection target of HL-RG and GC

A total of 105 overlapping targets between HL-RG and GSE65801 DEGs were identified, as shown in **Figure 1D**, among which 59 genes were upregulated and 46 were downregulated. We subsequently identified the intersections among the DEGs from the GEO database, the GC-related genes from the GeneCards and DisGeNET databases, and the targets of the active components of HL-RG, as depicted in **Figures 1E, F**. A total of 55 common targets were obtained, of which approximately two-thirds tended to be upregulated and one-third tended to be downregulated in the GC group. In other words, the 16 active components from HL-RG may exert their anti-GC effects through these 55 targets.

By ranking the log_2_FC values of these 55 intersection targets, we identified the genes with the most significant expression differences, as shown in **Figure 1G**. Notably, HOXC9, KLK8, OLFM4, FNDC1, and LIPF, along with ATP4A, GKN2, and GKN1, were among the top-ranked genes. Further analysis of the intersection targets was performed using principal coordinate analysis (PCoA), as depicted in **Figure 1H**, where red and green dots represent the normal and tumor groups, respectively, with minimal overlap between them.

### 3.3 Construction of a protein–protein interaction topology diagram

**Figure 2:**
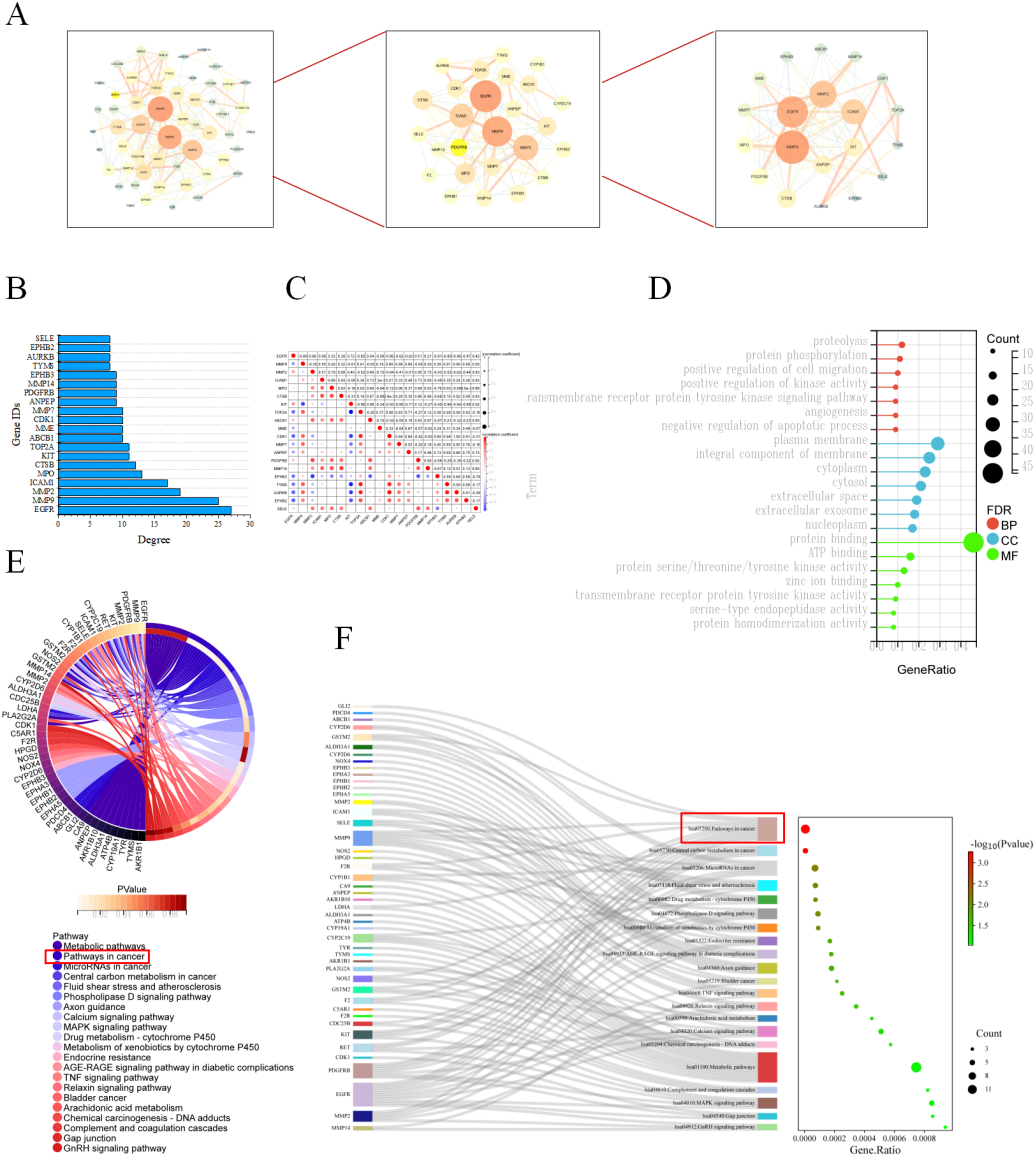
Screening and enrichment analysis of core targets. **(A)** Topology screening process of PPI network, the larger the circle and the redder the color, the more important the target is in the network. **(B)** Degree value of core targets. **(C)** Correlation heatmap of the core targets. Red represents positive correlation, blue represents negative correlation, and darker color indicates stronger correlation. **(D)** GO lollipop plot of intersecting targets. Red, blue and green represent BP, CC and MF respectively, the size of the circle represents the number of enriched targets, the bigger the circle, the more targets are enriched. **(E)** KEGG circle diagram of intersecting targets. The outermost left side of the circle represents the intersection target, the outermost right side color represents the pathway, and the color of the innermost right side of the circle represents the P-value of the enriched pathway; the lighter the color, the more significant the enriched pathway. **(F)** Ranking of the front KEGG Sankey diagram. The left side represents the intersection target, the middle side represents the pathway, the curve indicates the correlation between the two, and the rightmost bubble graph represents the result of KEGG, the larger the circle, the redder the color, the more significant the enriched pathway.

To further explore the key targets of HL-RG in the treatment of GC, 55 common targets were analyzed using Cytoscape software. As depicted in **Figure 2A**, the first screening was conducted with a degree cutoff of ≥ 7.25925925925925925, and the 20 targets obtained may represent the core targets of HL-RG in GC treatment. These included EGFR, MMP9, MMP2, ICAM1, MPO, CTSB, KIT, TOP2A, ABCB1, MME, CDK1, MMP7, ANPEP, PDGFRB, MMP14, EPHB3, TYMS, AURKB, EPHB2, and SELE.

The contribution of each target in the topological network was evaluated based on degree value, as shown in **Figure 2B**, with EGFR, MMP9, MMP2, and KIT ranking at the top. Correlation analysis revealed that EGFR and MMP2, as well as MMP2 and SERPINE1, were negatively correlated, whereas most of the other targets showed positive correlations, as illustrated in **Figure 2C**. These results indicate that the relationships among these targets are close and that their interactions are strong.

### 3.4 Bioinformatics analysis

To explore how the 55 common targets influence tumor initiation and progression, we first performed GO enrichment analysis. In the GO analysis (**Figure 2D**), BP, CC and MF terms are represented in red, blue, and green, respectively, with larger circles indicating a greater number of enriched targets. A total of 149 BP terms were enriched from the 55 targets, primarily related to phosphorylation, angiogenesis, apoptosis, and cell migration. A total of 33 CC terms were identified, revealing that the overlapping targets were predominantly localized in the cytosol, plasma membrane, extracellular space, and exosomes, suggesting their involvement in intercellular signal transduction. A total of 43 MF terms were obtained, indicating that the overlapping targets are mainly involved in regulating protein serine/threonine/tyrosine kinase activity, protein binding, transmembrane receptor protein tyrosine kinase activity, and serine-type endopeptidase activity.

**Figure 3:**
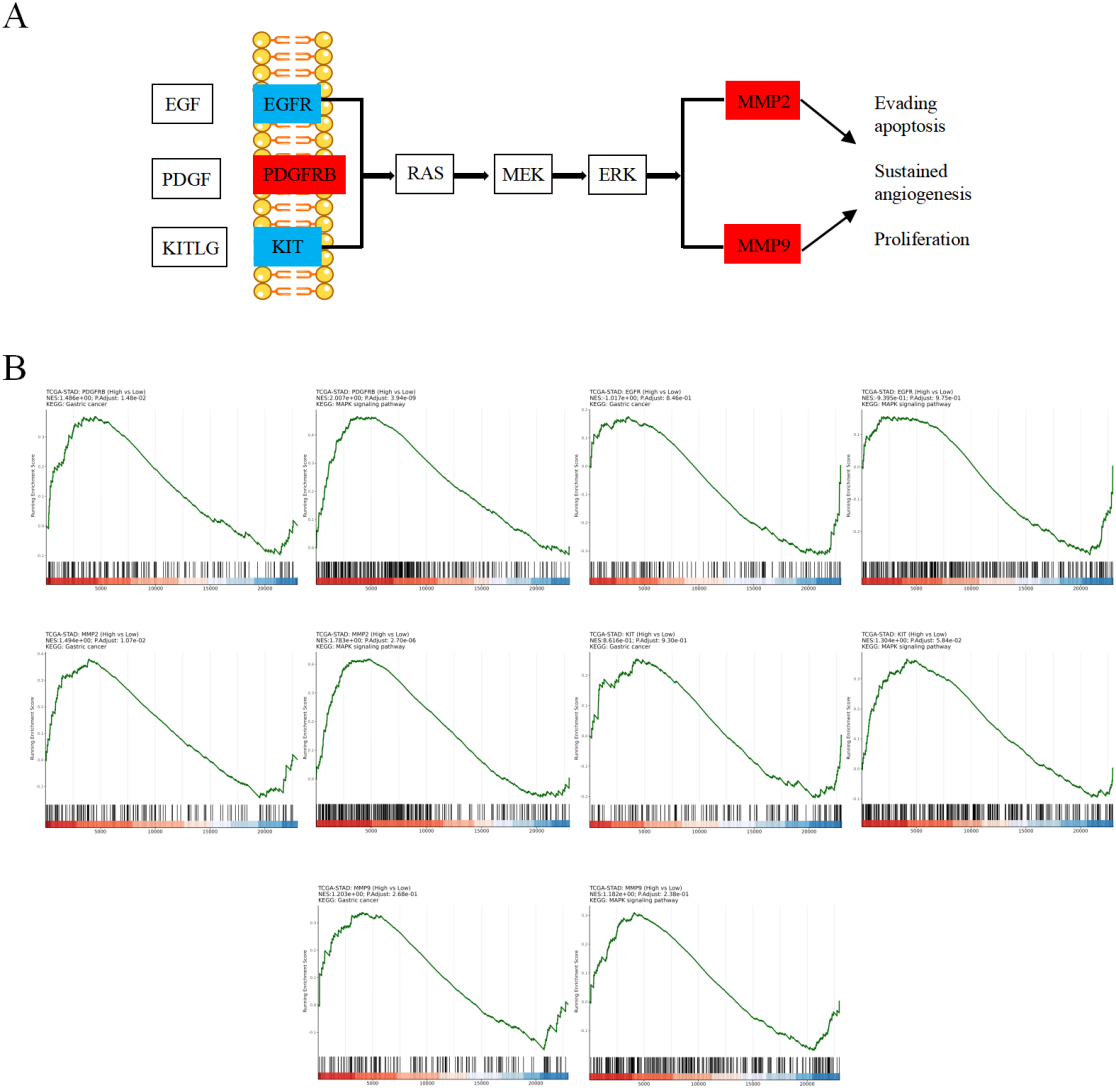
Screening and GSEA analysis of Hub genes. **(A)** Predicting and screening the role of Hub genes in the MAPK signaling pathway. Red nodes indicate up-regulation related genes and blue nodes indicate down-regulation related genes. **(B)** GSEA analysis graph of hub genes.

KEGG enrichment analysis was subsequently performed to explore the potential anti-GC mechanisms of HL-RG. A total of 21 signaling pathways were enriched from the 55 targets (**Figure 2E**). We further screened for core pathways based on the number of enriched targets. As shown in **Figure 2F**, Pathways in cancer ranked at the top. Upon closer inspection, we found that the MAPK signaling pathway contained the largest number of enriched intersection targets (**Figure 3A**). Within this pathway, the expression of MMP2, MMP9, and PDGFRB was upregulated, suggesting that the MAPK pathway may be crucial for the anti-GC effects of HL-RG. Accordingly, EGFR, PDGFRB, KIT, MMP2, and MMP9 were identified as hub genes.

We subsequently performed GSEA on all GC-related targets, as depicted in **Figure 3B**. The results revealed that the five hub genes were significantly correlated with GC and the MAPK signaling pathway. Moreover, as shown in **Figure 2D**, these five hub genes were associated with BP terms related to phosphorylation, angiogenesis, apoptosis, and migration, which is consistent with our previous analysis. Collectively, these findings suggest that HL-RG may regulate tumor cell biological processes such as migration and apoptosis through the MAPK signaling pathway, thereby exerting their pharmacological effects against GC.

### 3.5 Analysis of the clinical correlation of hub genes

**Figure 4:**
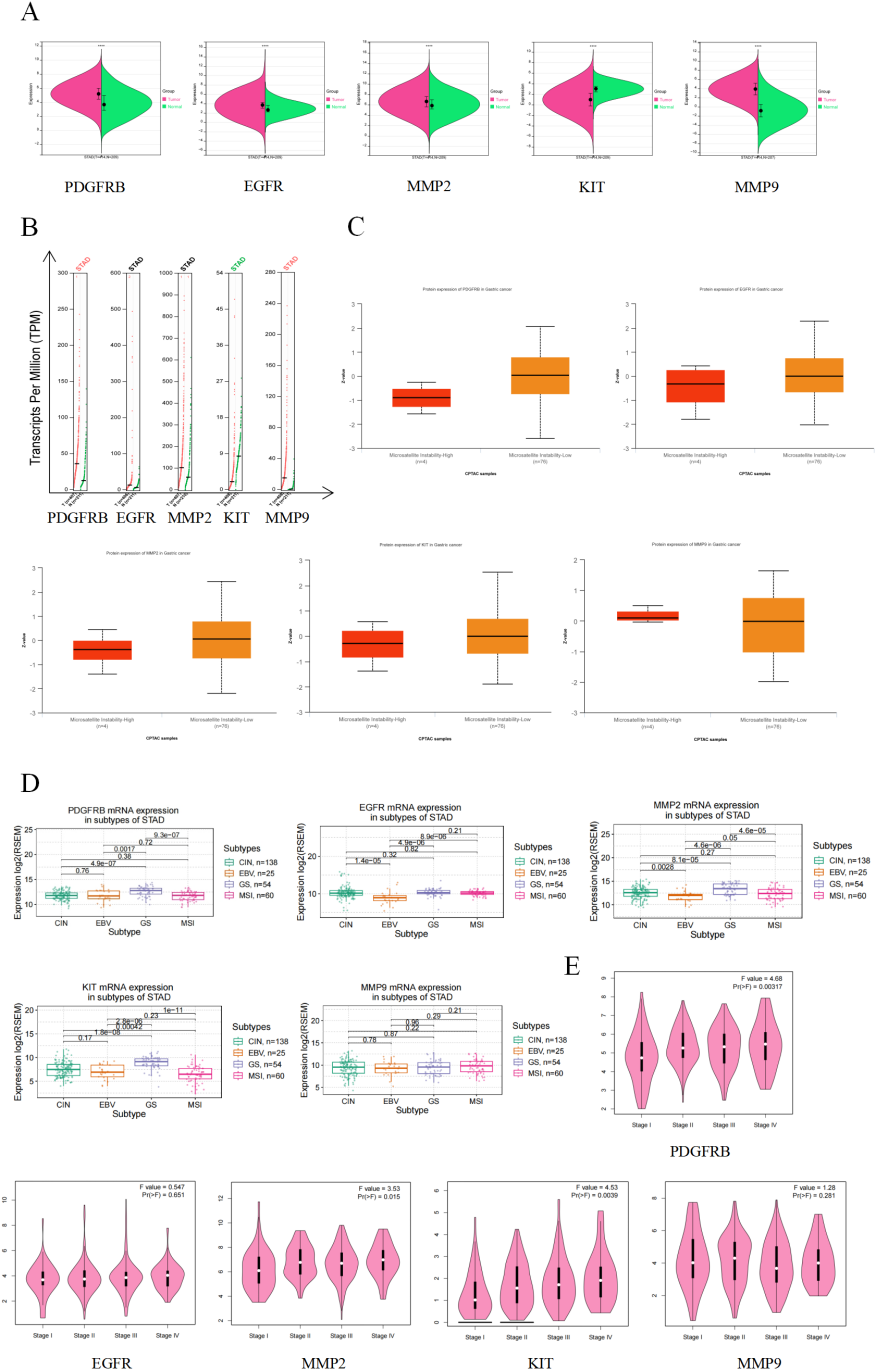
Clinical correlation analysis of hub gene. **(A)** Expression levels of hub gene mRNA. Red represents the tumor group and green represents the normal group. **(B)** Expression levels of hub gene copy number. Red represents the tumor group and green represents the normal group. **(C)** Expression level of hub gene protein. Red represents the microsatellite instability group and orange represents the microsatellite stability group. **(D)** Expression levels of hub gene in gastric cancer subtypes. **(E)** Correlation between hub gene and clinical stage of gastric cancer.

To explore the relationship between the hub genes and GC, we analyzed their expression profiles at both the transcriptional and translational levels. As illustrated in **Figures 4A-C**, the five hub genes were highly expressed in GC at the mRNA level. At the copy number level, PDGFRB and MMP9 also showed high expression in GC. However, at the protein level, we observed low expression of PDGFRB, KIT, MMP2, and EGFR in the microsatellite stable (MSS) subtype of GC, whereas MMP9 exhibited high expression. The differences in gene expression among GC subtypes are shown in **Figure 4D**. The five hub genes were highly expressed in the CIN subtype but expressed at low levels in the MSI subtype.

**Figure 5:**
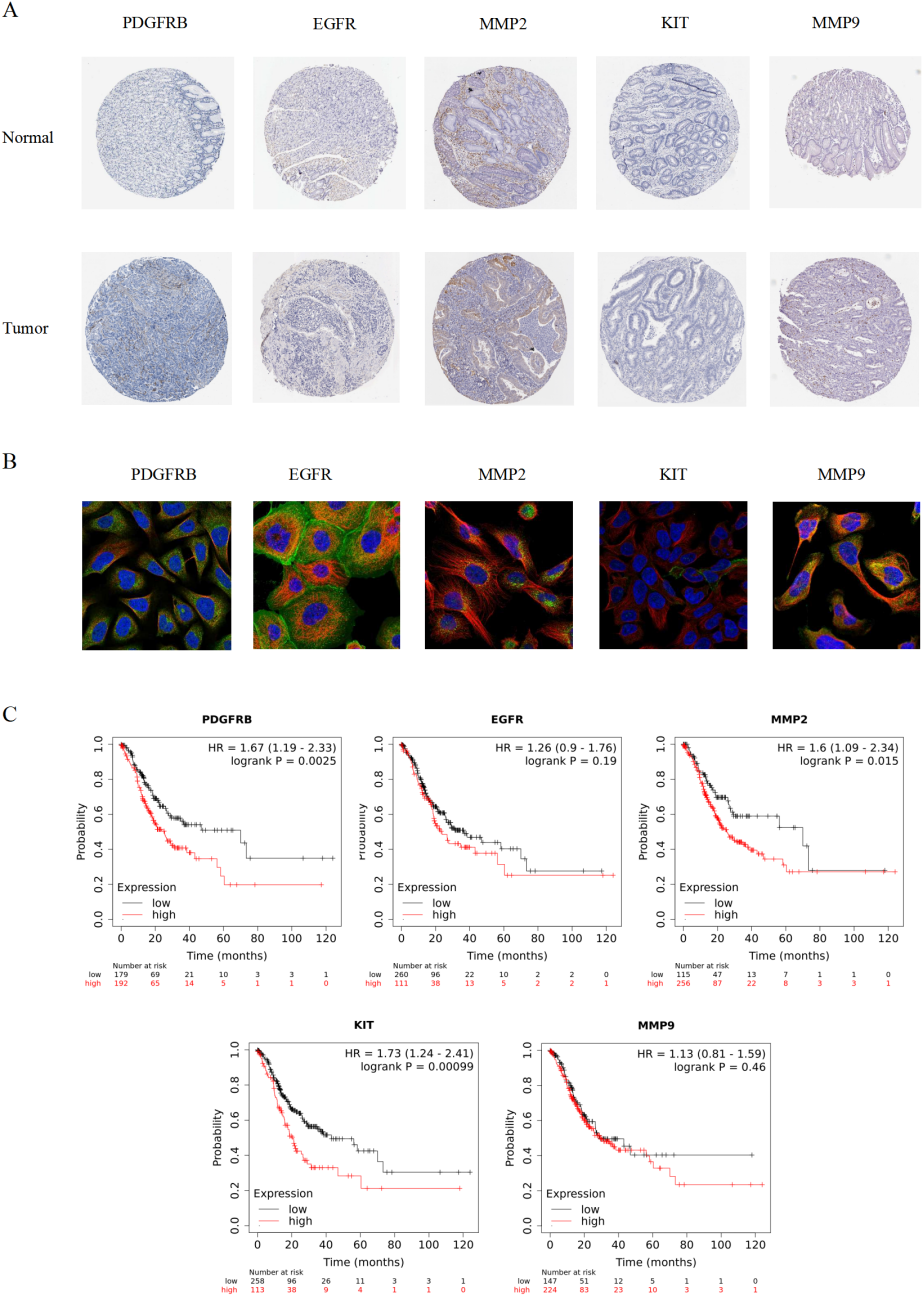
Expression and prognostic analysis of hub gene. **(A)** Immunohistochemistry of Hub gene in normal gastric tissue and gastric adenocarcinoma tissue. Brown color shows the expression level of Hub gene. **(B)** Fluorescence localization map of Hub gene in tumor tissues. Blue represents nucleus, red represents microtubule organization, and green represents Hub gene. **(C)** Survival curve diagram of Hub gene. The horizontal coordinate represents the survival time.

We further analyzed the correlation between hub gene expression and the clinical stage of GC. As shown in **Figure 4E**, MMP2 expression increased in middle and advanced stages of GC. Given that expression trends at the transcriptional and translational levels do not necessarily align, we further investigated the protein expression of the hub genes using the HPA database. As illustrated in **Figure 5A**, the brown staining in the immunohistochemical images indicates regions of elevated protein expression. All five hub genes showed markedly increased expression in GC tissues, which is consistent with our clinical correlation results.

To better understand the subcellular localization of these hub genes in tumor tissues, we obtained immunofluorescence images from the HPA database. As displayed in **Figure 5B**, blue fluorescence marks the cell nucleus, red indicates microtubular structures, and green shows the localization of the hub genes. Notably, MMP9 was expressed primarily in vesicles, while both MMP9 and another gene showed increased expression in the cytoplasm. In contrast, EGFR was expressed predominantly in the cell membrane and at cell junctions.

The prognostic significance of the hub genes is illustrated in **Figure 5C**. With the exception of EGFR and MMP9, the expression of MMP2, KIT, and PDGFRB was significantly associated with the overall survival (OS) of GC patients.

### 3.6 Mutation and methylation of the hub genes are associated with GC

**Figure 6:**
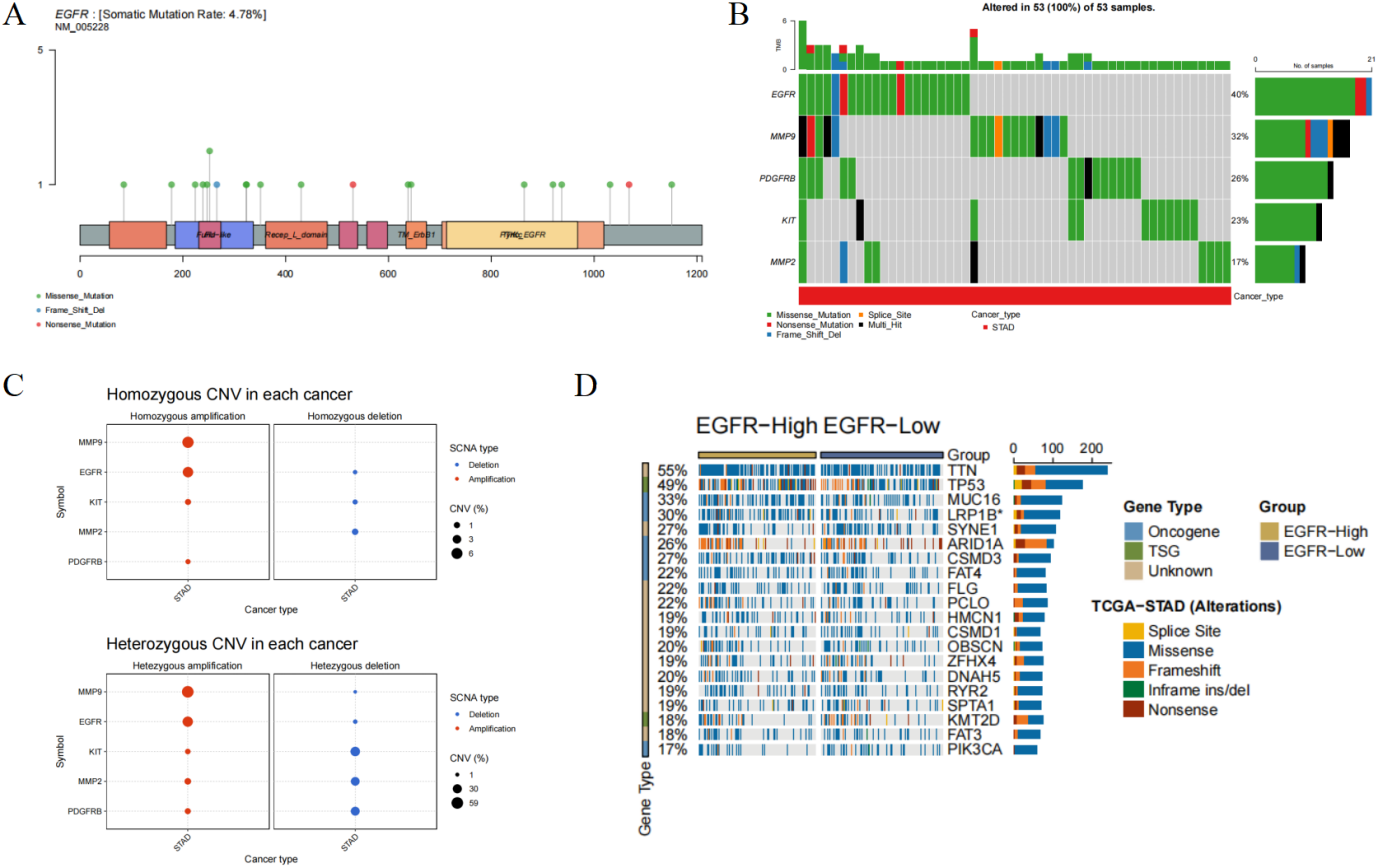
Effect of mutations in the Hub gene on gastric cancer. **(A)** SNV mutation sites and types of EGFR. The circle color represents the mutation type and the line length represents the mutation frequency. **(B)** Waterfall plot of SNV mutation frequency of Hub gene. The upper bar indicates the proportion of Hub gene mutations in the 53 samples. **(C)** Bubble plots of heterozygous and pure heterozygous CNV mutations in the Hub gene. The larger the bubble, the higher the proportion of mutations. **(D)** Mutational associations of driver genes with the Hub gene.

Tumor occurrence is characterized by genomic alterations. As shown in **Figure 6A**, the predominant type of single nucleotide variant (SNV) among the hub genes was “missense mutation.” We further analyzed the frequency of deleterious mutations in the hub genes (**Figure 6B**). EGFR had the highest mutation frequency, accounting for 40% of mutations, followed by MMP9 (32%), while MMP2 had the lowest frequency at only 17%.

Copy number variations (CNVs) include both heterozygous and homozygous mutations, with the latter causing more pronounced disease consequences. As illustrated in **Figure 6C**, bubbles are proportional in size to mutation frequency, with red representing amplification and blue representing deletion. We observed that elevated EGFR expression was associated with increased expression of oncogenes such as MUC1 (**Figure 6D**).

Since DNA methylation occurs in some tumors, altering chromatin structure, DNA conformation, and stability, and regulating gene transcription, we analyzed the correlation between methylation and the five hub genes. As shown in **Supplementary Figure 1A**, KIT and MMP9 showed low expression after methylation in the GC group, whereas PDGFRB, EGFR, and MMP2 were highly expressed. Additionally, hub gene methylation was positively correlated with both T lymphocyte-producing cells and survival risk (**Supplementary Figures 1B, C**).

Genetic mutations are often closely associated with the occurrence and progression of GC, whereas genomic stability relies on various repair mechanisms, including mismatch repair (MMR) and homologous recombination repair (HRR). Consequently, we performed a correlation analysis between repair system genes and hub gene mutations (**Supplementary Figures 1D, E**). In these figures, the vertical axis represents repair system-related genes, the horizontal axis represents hub genes, and red and green indicate positive and negative correlations, respectively, with darker colors indicating stronger correlations. We found that MMP9 and EGFR were significantly positively correlated with the HRR repair system, while PDGFRB, MMP2, and KIT were negatively correlated with HRR. In the MMR repair system, repair-related genes were positively correlated with all five hub genes, with PDGFRB, EGFR, KIT, and MMP9 showing the most significant differential expression.

### 3.7 Bioinformatics prediction of immune correlations

To explore potential links between hub genes and the tumor immune microenvironment, we performed bioinformatics analyses using CIBERSORT, TISCH2, and other public databases. As shown in **Supplementary Figures 2A-E**, these analyses predicted that the hub genes may correlate with certain immune cell populations (e.g., NK cells, macrophages), immune checkpoint molecules, and stromal/immune scores. However, these findings are based solely on computational predictions and have not been experimentally validated. Functional studies (e.g., T cell−GC cell co−culture or immune−competent animal models) are required to determine whether HL−RG actually modulates immune responses in GC.

### 3.8 Drug therapy

**Figure 7:**
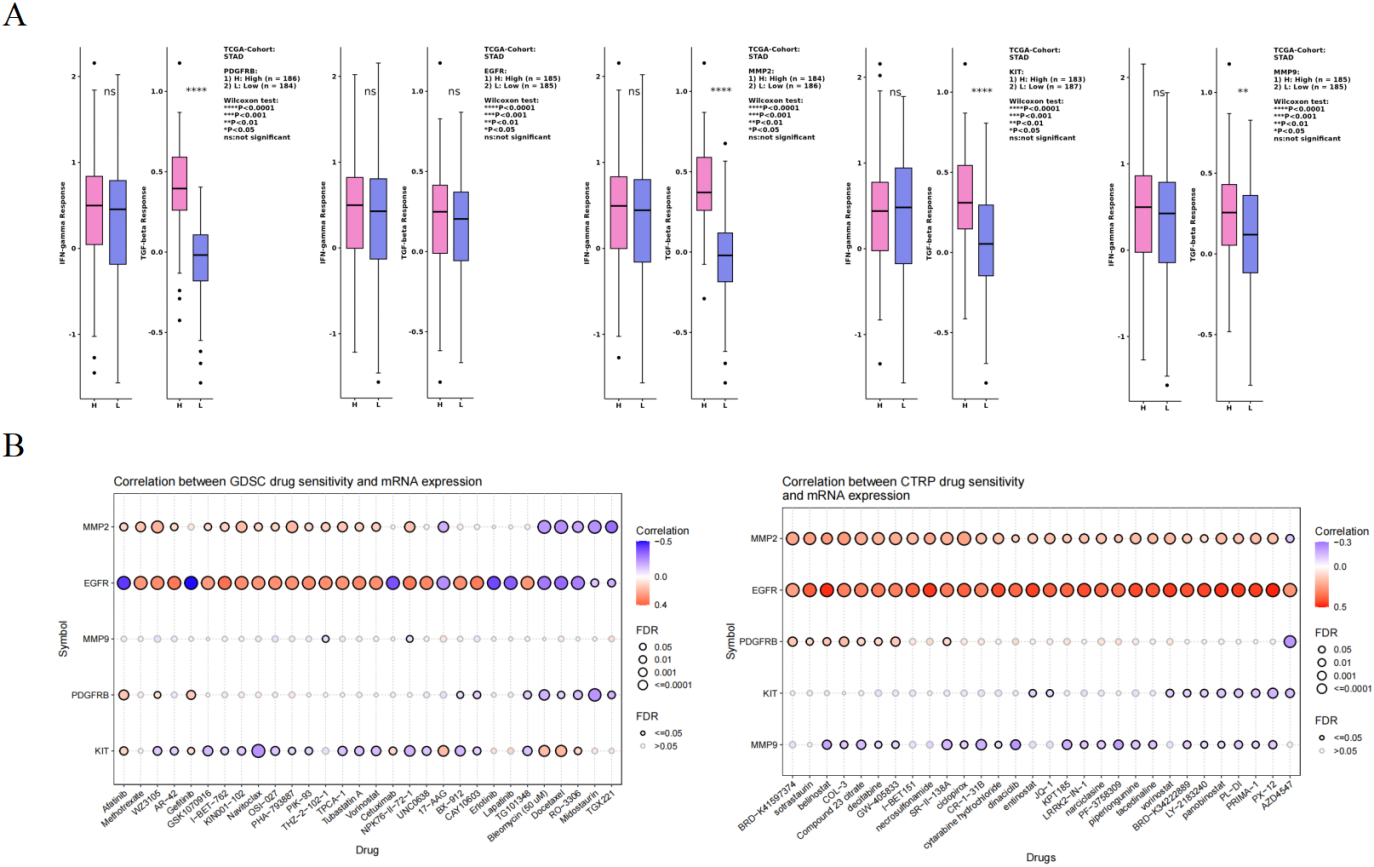
Association of hub genes with antitumor drug therapy. **(A)** Association of 5 hub gene expression levels with IFN-γ, TGF-β. **(B)** Correlation of hub genes with immunotherapy.

The TISIDB website was used to analyze the correlations between the hub genes and immunotherapy response. Given that IFN-γ can enhance the killing effect of drugs on tumor cells, while TGF-β induces tumor cell apoptosis and inhibits their proliferation, the correlations between the hub genes and these two cytokines were calculated using the CAMOIP website. Additionally, the drug sensitivity of the hub genes to antitumor drugs was analyzed using the GSCA database, and the results were visualized as bubble plots (**Figures 7A, B**).

### 3.9 A core component–core target network diagram and molecular docking were constructed

**Figure 8:**
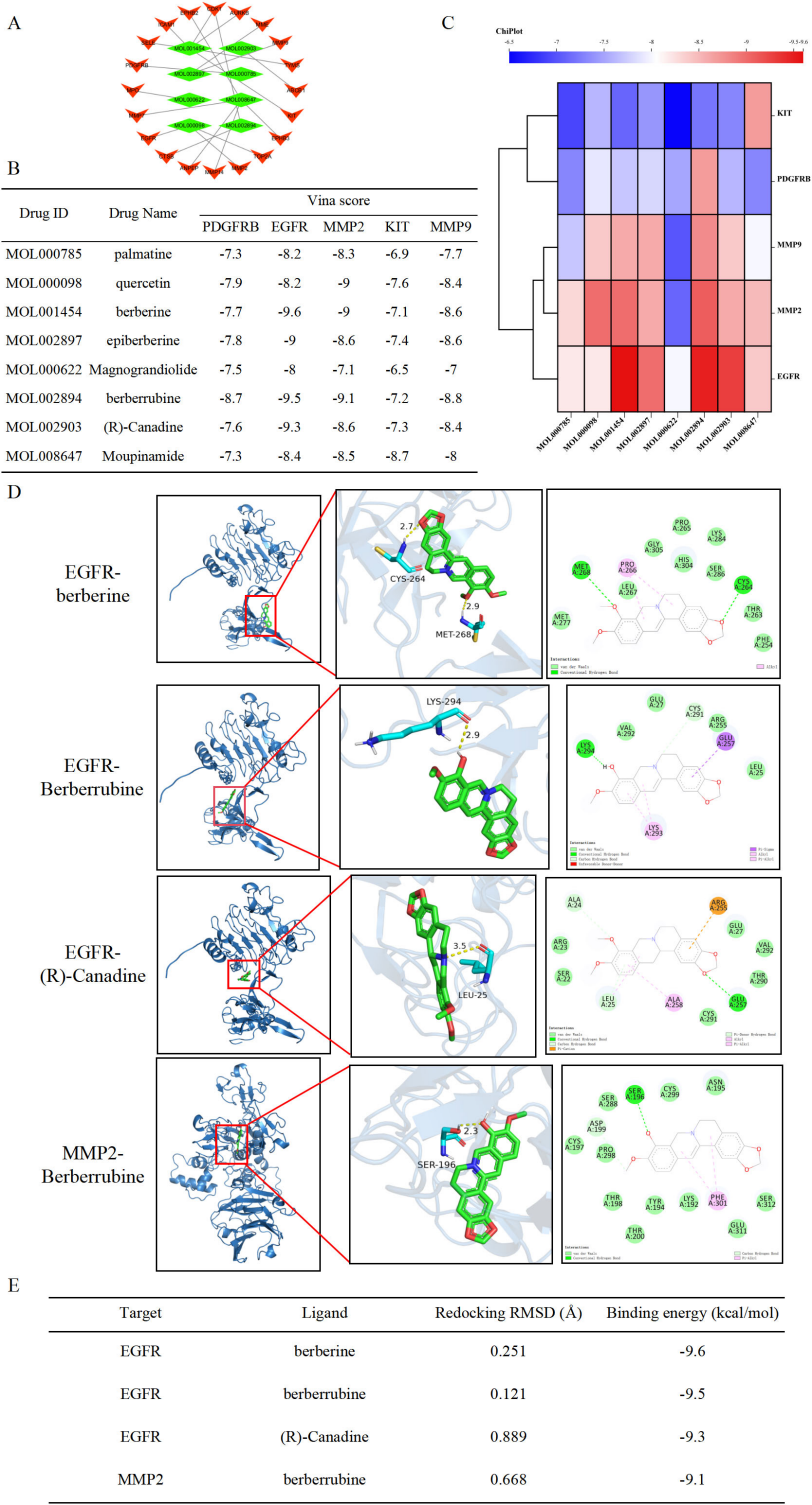
Molecular docking of hub genes with core active ingredients. **(A)** Screening of core active ingredients exerting anti-gastric cancer therapeutic effects, with red triangles representing the targets and green rhombuses representing the Drug ID of the active ingredients. **(B, C)** Molecular docking binding energy of hub genes and core active ingredients. **(D)** Visual demonstration of partial molecular docking. **(E)** Redocking validation of HL-RG components with EGFR and MMP2.

To further explore the core active components and specific targets of HL-RG in the treatment of GC, we constructed a network diagram of the 20 hub targets and their corresponding components using Cytoscape. As shown in **Figure 8A**, MOL000785 (palmatine), MOL000098 (quercetin), MOL001454 (berberine), MOL002897 (epiberberine), MOL000622 (magnograndiolide), MOL002894 (berberrubine), MOL002903 ((R)-canadine), and MOL008647 (mogupinamide) may be the key active components of HL-RG that play antitumor roles.

Molecular docking experiments can predict the binding affinity between the active components of HL-RG and the GC targets, thereby simulating drug absorption and metabolism in the human body. As illustrated in **Figures 8B, C**, the binding energies of MOL000785, MOL000098, MOL001454, MOL002897, MOL000622, MOL002894, MOL002903, and MOL008647 with the five hub genes were all < -6.5 kcal/mol. Among them, the binding energies of EGFR with MOL001454, MOL002894, and MOL002903, as well as MMP2 with MOL002894, were the lowest, with values of -9.6, -9.5, -9.3, and -9.1 kcal/mol, respectively. The molecular docking results were visualized, as shown in **Figure 8D**.

To verify the reliability of our molecular docking parameters, we performed redocking experiments using the co-crystallized ligands of EGFR and MMP2. The re-docked poses showed excellent agreement with the original crystal structures, with RMSD values of 0.251 Å (EGFR-berberine), 0.121 Å (EGFR-berberrubine), 0.889 Å (EGFR-(R)-canadine), and 0.668 Å (MMP2-berberrubine) (**Figure 8E**). All RMSD values were below the acceptable threshold of 2.0 Å, confirming that our docking protocol can accurately reproduce the experimental binding modes. Therefore, the docking scores (binding energies) for the other HL-RG components are considered reliable for predicting potential interactions.

### 3.10 HL-RG inhibits the proliferation of GC

**Figure 9:**
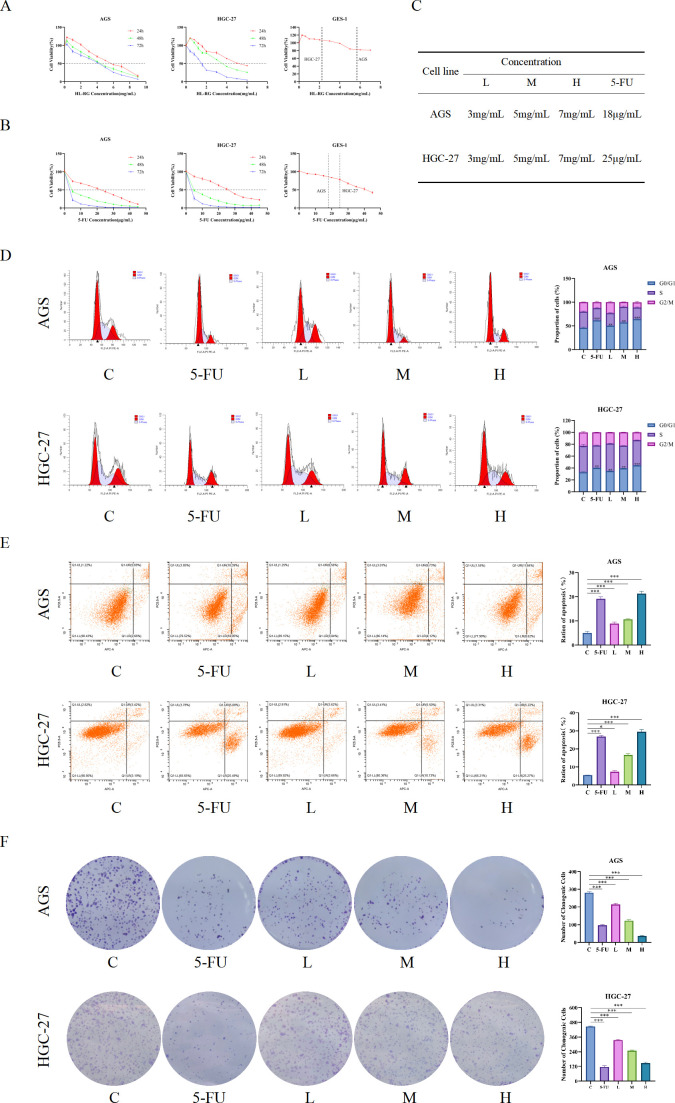
Proliferative phenotype of gastric cancer inhibited by HL-RG. **(A)** AGS and HGC-27 cells were treated with different concentrations of HL-RG for 24 h, 48 h and 72 h. **(B)** AGS and HGC-27 cells were treated with different concentrations of 5-FU for 24 h, 48 h and 72 h. **(C)** IC_50_ values for 24, 48 h and 72 h of AGS and HGC-27 cells. **(D)** Effect of HL-RG and 5-FU on the cell cycle of AGS and HGC-27 cells. **(E)** Effect of HL-RG and 5-FU on the apoptosis rate in AGS and HGC-27 cells. **(F)** Effect of HL-RG and 5-FU on the number of colony formation in AGS and HGC-27 cells. All data are presented as mean ± SD from three independent biological replicates (n=3). Statistical significance was determined by Student’s t test (two groups) or one-way ANOVA (three or more groups).*P<0.05,**P<0.01, ***P<0.001.

To evaluate the effect of HL-RG on the viability of gastric cancer cells, we performed CCK-8 assays to detect cell proliferation. The results showed that HL-RG inhibited the growth of gastric cancer cells in a time and dose dependent manner. After treatment with HL-RG for 24, 48, and 72 h, the IC50 values for AGS cells were 5.680, 4.357, and 3.720 mg/mL, respectively, and those for HGC-27 cells were 2.251, 1.890, and 1.318 mg/mL, respectively (**Figure 9A**). We selected the classic anticancer drug 5-FU as a positive control. Based on the CCK-8 results, the effective concentrations for AGS and HGC-27 cells were determined to be 18 μg/mL and 25 μg/mL, respectively (**Figure 9B**). Moreover, compared with normal gastric mucosal GES-1 cells, the survival rate of GC cells treated with HL-RG was greater than 80% at doses of 5 and 7 mg/mL. Therefore, low-, medium-, and high-dose groups treated with 3, 5, and 7 mg/mL HL-RG were selected for subsequent experiments (**Figure 9C**).

Subsequently, we performed cell cycle, apoptosis, and colony formation assays on gastric cancer cells. Flow cytometry analysis revealed that the percentages of AGS cells in the G0/G1 phase in the low-, medium-, and high-dose groups were 50.71%, 58.77%, and 63.54%, respectively, while the 5-FU group exhibited a value of 62.75%. All these values were significantly higher than that of the control group. Similarly, the proportions of HGC-27 cells in the G0/G1 phase were 34.67%, 39.56%, and 44.58% in the HL-RG groups, and 40.05% in the 5-FU group, all significantly increased compared with the control (**Figure 9D**).

Apoptosis was also assessed by flow cytometry. The apoptotic rates of AGS cells in the low-, medium-, and high-dose HL-RG groups were 9.60%, 10.85%, and 21.46%, respectively, while the 5-FU group showed a rate of 20.44%. For HGC-27 cells, the apoptosis rates were 7.28%, 16.23%, and 30.49% in the HL-RG groups, and 30.57% in the 5-FU group. Compared with the control group, the apoptosis rates of GC cells were significantly elevated (**Figure 9E**).

Finally, the proliferative capacity of the gastric cancer cell lines AGS and HGC-27 was evaluated via a colony formation assay. The number of crystal violet-stained colonies in the 5-FU group and all HL-RG treatment groups was significantly reduced compared with the control group, indicating a lower colony formation rate (**Figure 9F**). Taken together, these results demonstrate that HL-RG effectively inhibits the proliferation of GC cells.

**Figure 10:**
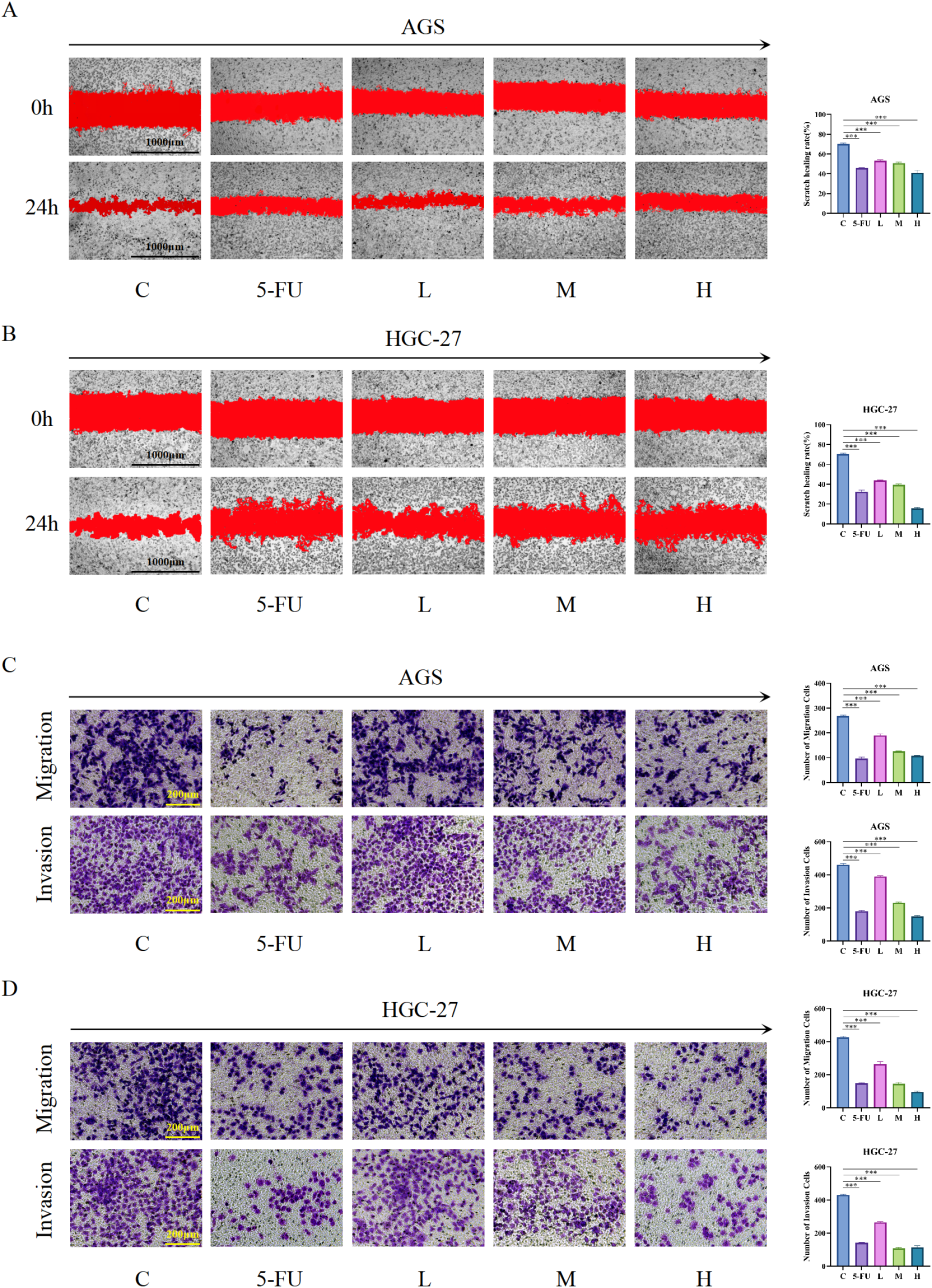
Metastatic phenotype of gastric cancer inhibited by HL-RG. **(A, B)** Effect of HL-RG on horizontal migration of AGS and HGC-27 cells after 24h intervention. Scale bars = 1000 µm. **(C, D)** Effect of HL-RG on vertical migration of AGS and HGC-27 cells after 24h intervention. Scale bars = 200 µm. All data are presented as mean ± SD from three independent biological replicates (n=3). Statistical significance was determined by Student’s t test (two groups) or one-way ANOVA (three or more groups). *P<0.05, **P<0.01, ***P<0.001.

### 3.11 HL-RG inhibits the metastasis of GC

To further confirm whether HL-RG can inhibit the metastatic capacity of gastric cancer cells, we conducted wound healing, migration, and invasion assays. After scratching AGS and HGC-27 cell monolayers, the cells were treated with low, medium, or high doses of HL-RG and 5-FU, and wound healing was observed at 0 and 24 hours. Analysis showed that, compared with the control group, the wound healing areas in the HL-RG-treated and 5-FU-treated groups were significantly smaller (**Figures 10A, B**).

The results of the Transwell assay were consistent with the above findings. Following treatment with different doses of HL-RG and 5-FU, the migration and invasion capabilities of AGS and HGC-27 cells were significantly reduced in a dose-dependent manner (**Figures 10C, D**). In summary, these results indicate that HL-RG effectively inhibits the metastasis of gastric cancer cells.

### 3.12 Effects of HL-RG on the expression of hub genes

**Figure 11:**
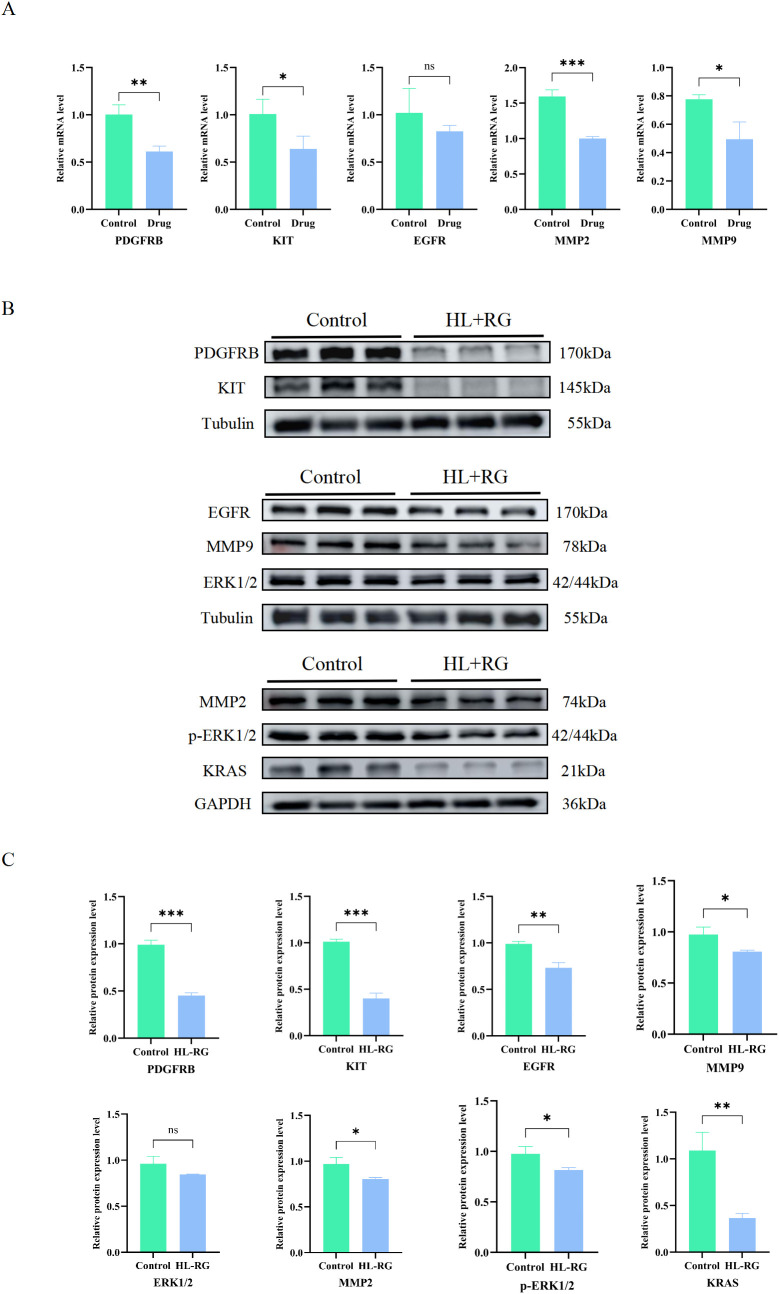
HL-RG regulated hub genes and MAPK signaling pathway. **(A)** mRNA level of 5 hub genes in AGS cells treated with HL-RG for 24 h. **(B, C)** Level of key proteins in MAPK signaling pathway after HL-RG treatment of AGS cells for 24 h. All data are presented as mean ± SD from three independent biological replicates (n=3). Statistical significance was determined by Student’s t test (two groups) or one-way ANOVA (three or more groups). *P<0.05, **P<0.01, ***P<0.001.

Through network pharmacology analysis, we identified five key genes: PDGFRB, EGFR, MMP2, MMP9, and KIT that may play a critical role in the anti-GC effects of HL-RG. Analysis of public databases indicated that these five key genes exhibit significant differential expression in gastric cancer and are associated with tumor proliferation and metastasis. Therefore, we used qRT-PCR to detect the expression of these key genes. Compared with the control group, the expression levels of PDGFRB, KIT, MMP2, and MMP9 were significantly reduced in AGS cells treated with HL-RG, while no significant change was observed in EGFR expression (**Figure 11A**). These results suggest that these five key genes may be the core targets mediating the anticancer effects of HL-RG against GC.

KEGG and GSEA enrichment analyses revealed that these key genes are significantly associated with the MAPK signaling pathway. To validate this hypothesis, we examined the expression levels of key proteins in the MAPK pathway (including KRAS, ERK, and phosphorylated ERK (p-ERK)) via Western blotting. The results were consistent with the qRT-PCR findings. Following HL-RG treatment, the expression levels of KRAS and p-ERK1/2 decreased significantly, while the total ERK1/2 levels showed no significant change (**Figures 11B, C**). These results suggest that HL-RG may inhibit the proliferation and metastasis of gastric cancer cells through the RAS/RAF/MEK/ERK signaling pathway.

### 3.13 MAPK pathway rescue experiments

To establish the causal relationship between HL−RG treatment and MAPK pathway activation, we performed rescue experiments using the MEK inhibitor U0126 and the MAPK agonist EGF.

**Figure 12:**
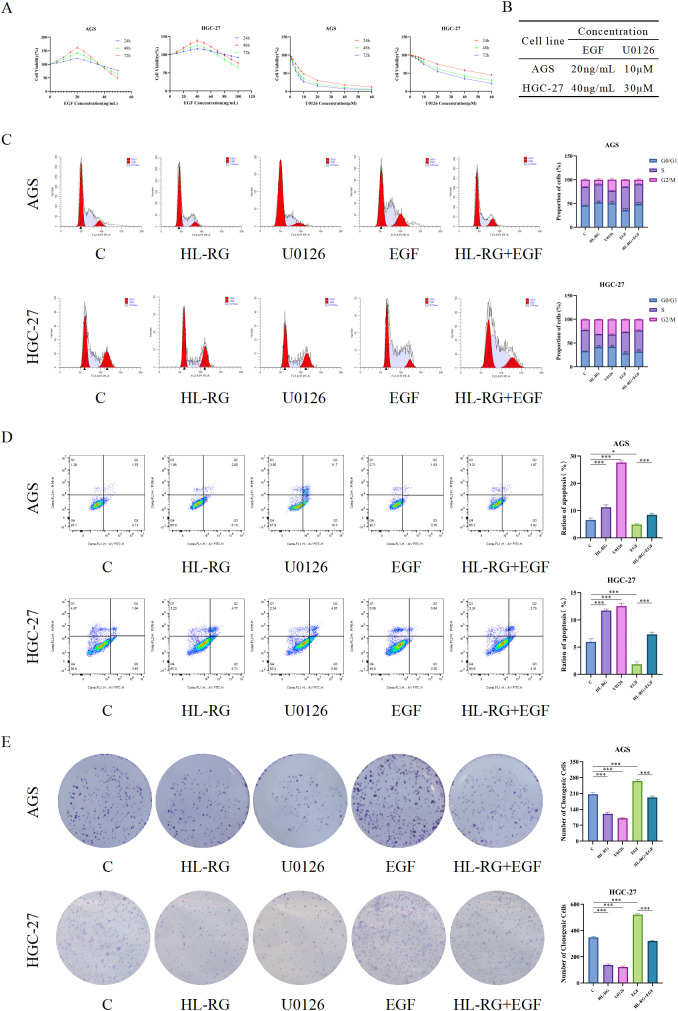
MAPK rescue experiments on proliferation. **(A)** AGS and HGC-27 cells were treated with different concentrations of EGF and U0126 for 24, 48, and 72 hours. **(B)** Concentrations of EGF and U0126 used in AGS and HGC-27 cells. **(C)** Effect of HL-RG, U0126, EGF, and HL-RG+EGF on the cell cycle of AGS and HGC-27 cells. **(D)** Effect of HL-RG, U0126, EGF, and HL-RG+EGF on the apoptosis rate in AGS and HGC-27 cells. **(E)** Effect of HL-RG, U0126, EGF, and HL-RG+EGF on the number of colony formation in AGS and HGC-27 cells. All data are presented as mean ± SD from three independent biological replicates (n=3). Statistical significance was determined by Student’s t test (two groups) or one-way ANOVA (three or more groups). *P<0.05, **P<0.01, ***P<0.001.

Proliferation phenotypes: In both AGS and HGC−27 cells, EGF treatment significantly reversed the HL−RG−induced G0/G1 phase arrest (**Figure 12C**), increase in apoptosis (**Figure 12D**), and reduction of colony formation (**Figure 12E**), Conversely, U0126 alone phenocopied the effects of HL−RG on these proliferation−related phenotypes. The CCK−8 dose−response curves are shown in **Figure 12A**, and the concentrations of EGF and U0126 used are summarized in **Figure 12B**.

**Figure 13:**
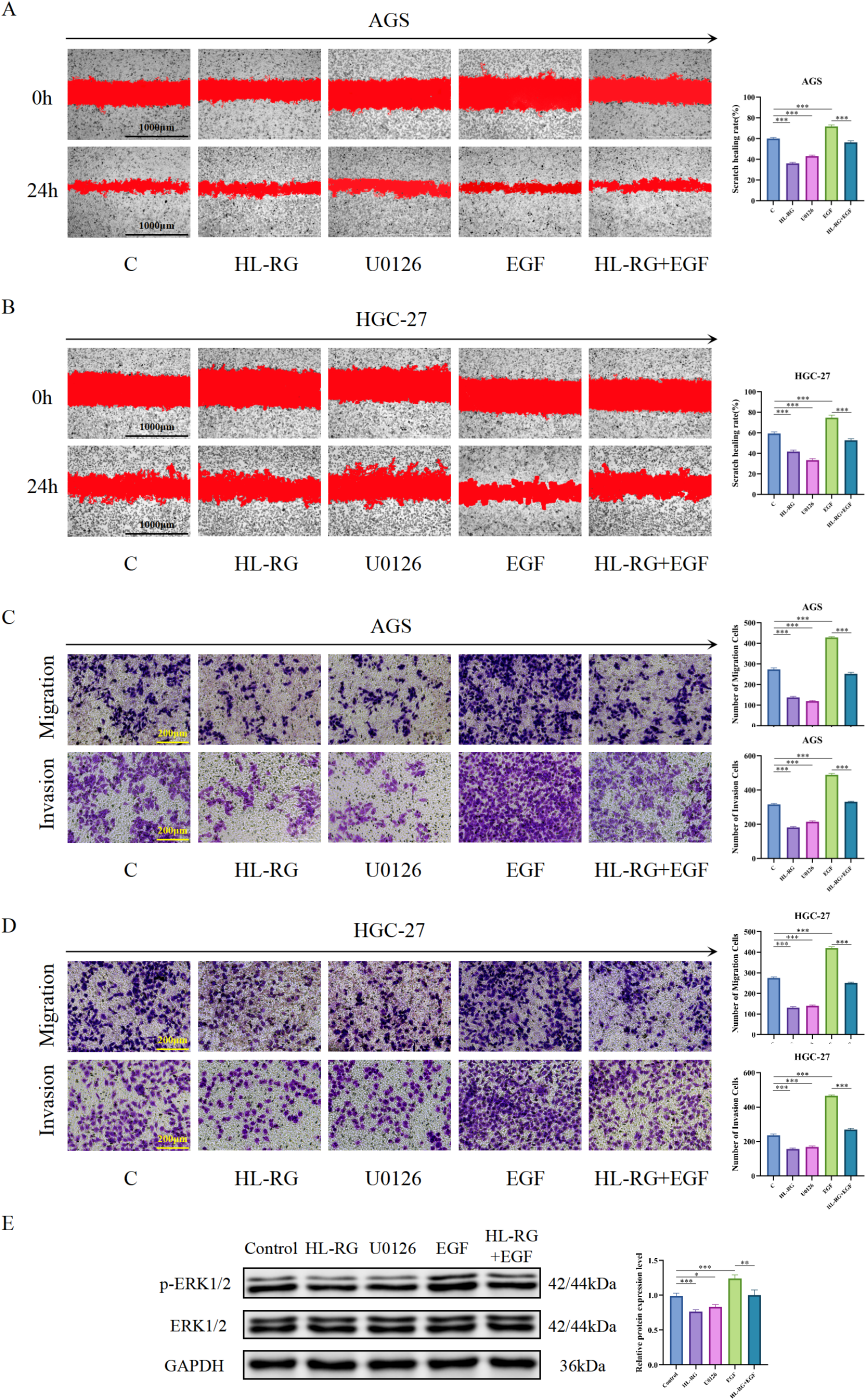
MAPK rescue experiments on metastasis and signaling. **(A, B)** Effect of HL-RG, U0126, EGF, and HL-RG+EGF on horizontal migration of AGS and HGC-27 cells after 24h intervention. Scale bars = 1000 µm. **(C, D)** Effect of HL-RG, U0126, EGF, and HL-RG+EGF on vertical migration of AGS and HGC-27 cells after 24h intervention. Scale bars = 200 µm. **(E)** Western blotting analysis the protein expression levels of ERK1/2 and p-ERK1/2 in gastric cancer cells.All data are presented as mean ± SD from three independent biological replicates (n=3). Statistical significance was determined by Student’s t test (two groups) or one-way ANOVA (three or more groups). *P<0.05, **P<0.01, ***P<0.001.

Metastatic phenotypes: Wound healing assays (**Figures 13A, B**) and Transwell migration/invasion assays (**Figures 13C, D**) demonstrated that EGF rescued the migratory and invasive capacities of HL−RG−treated GC cells, while U0126 alone suppressed migration and invasion similarly to HL−RG.

Molecular evidence (**Figure 13E**): Western blot analysis showed that HL−RG reduced p−ERK1/2 levels without affecting total ERK1/2. EGF alone increased p−ERK1/2 expression, and co−treatment with EGF partially restored p−ERK1/2 levels compared to HL−RG alone. U0126 alone markedly reduced p−ERK1/2 expression.

Collectively, these results demonstrate that HL−RG suppresses GC cell proliferation, induces apoptosis, and inhibits migration and invasion through the MAPK/ERK signaling pathway.

## 4 Discussion

GC is a major global health concern, ranking as the fifth most prevalent cancer and the fifth leading cause of cancer-related deaths worldwide (31). Despite the diversification of treatment strategies for GC, the prognosis for patients with advanced GC remains poor (32). Hence, there is an urgent need to explore new directions in GC treatment to improve diagnosis, therapy, and patient prognosis.

In the early stage of GC, there are usually no specific symptoms, or symptoms are mild and easily confused with common gastric diseases such as gastritis and gastric ulcers (32). Patients may only experience minor upper abdominal discomfort or appetite changes, which are often mistaken for temporary discomfort and ignored; consequently, most patients are diagnosed at middle or late stages. Surgery is currently the only curative treatment for localized disease. In patients with metastatic disease, survival is significantly reduced, and the treatment of choice is often palliative chemotherapy with or without radiotherapy. Although radiotherapy and chemotherapy can inhibit tumor growth and metastasis to a certain extent in non-surgical patients, the resulting side effects and drug resistance limit their clinical use (33, 34).

To date, numerous studies have shown that TCM is playing an increasingly important role in clinical therapy and preventive healthcare (35). TCM has the advantages of multiple targets, multiple pathways, and multiple effects in the treatment of GC and is an indispensable part of the comprehensive treatment system for GC (35). It is highly important to further research the effectiveness of TCM in the clinical treatment of gastric cancer, comprehensively explore the molecular mechanisms of its action, and identify new therapeutic targets for improving GC treatment (35).

HL-RG based formulations have been shown to be effective in treating digestive diseases (36). The Guilian Pill, which is composed of HL-RG, originates from TCM formulas and is used to treat gastrointestinal disorders (36). Documented in the Treatise on Febrile Diseases by Zhang Zhongjing in the Han Dynasty, related formulations have been well documented in ancient Chinese medical books for their ability to control gastrointestinal diseases. Modern scientific research has confirmed their effectiveness in the treatment of gastric cancer (37). Studies have confirmed that the active ingredients of HL-RG can effectively inhibit the proliferation, invasion, and migration of gastric cancer cells through multiple mechanisms, including regulation of the cell cycle, cell differentiation, and metastasis (38).

**Figure 14:**
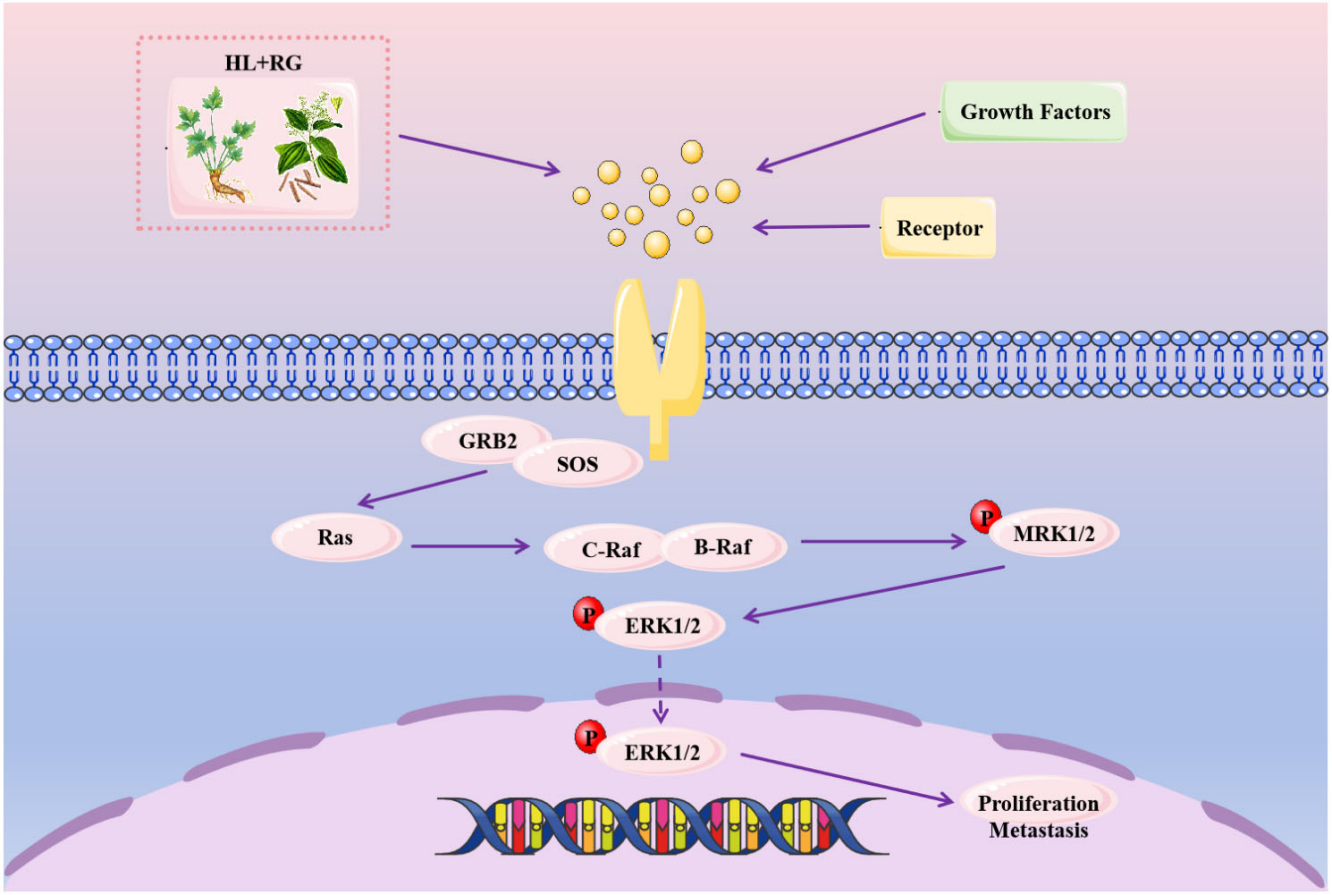
Mechanism diagram of HL-RG anti-gastric cancer.

**Figure 14** illustrates the anti‑gastric cancer mechanism of HL‑RG. In this study, we used network pharmacology, bioinformatics, and *in vitro* cellular functional verification to explore the targets and pharmacological mechanisms of HL-RG in inhibiting GC (39). First, we obtained the potential active constituents of HL and RG from the TCMSP database. Palmatine, quercetin, berberine, epiberberine, magnograndiolide, berberrubine, (R)-canadine, and moupinamide were identified as key components. After intersecting gastric cancer and drug targets, we identified five hub genes: PDGFRB, EGFR, MMP2, KIT, and MMP9, which may be the main targets of the antitumor effects of HL-RG. Recent studies on natural alkaloids have demonstrated that berberine (a key component of HL) can disrupt key signaling pathways in cancer cells, including MAPK and EGFR (40). Molecular docking predicted strong binding affinities between HL−RG components and hub genes, with binding energies below -9.0 kcal/mol. Redocking validation gave RMSD values < 2.0 Å, confirming the reliability of our docking protocol. However, direct biophysical assays are needed to validate physical binding in the future.

To further explore the correlation between these five hub genes and gastric cancer, we examined their mRNA expression, transcriptional regulation, translational levels, and epigenetic modifications using public databases (41). Our findings revealed significant differences in their expression patterns in GC, suggesting that altered expression of these genes leads to pronounced abnormalities in cancer cells and tissues, thereby influencing tumor growth and proliferation rates (42). Moreover, these five hub genes were related to the stage and prognosis of gastric cancer patients, providing evidence supporting clinical treatment selection and prognosis prediction (43).

In bioinformatics analysis, we found that PDGFRB, KIT, MMP2, and EGFR were expressed at lower levels in the microsatellite−stable (MSS) subtype of gastric cancer compared to other subtypes. This observation has potential clinical implications for HL−RG therapy. MSS gastric cancer is generally less responsive to immune checkpoint inhibitors and has a distinct tumor microenvironment. The reduced expression of these hub genes in MSS tumors suggests that HL−RG, which targets these genes, might exhibit subtype−specific efficacy. Specifically, patients with MSS subtype GC could be less likely to benefit from HL−RG if its mechanism depends on downregulating these targets; conversely, if HL−RG acts upstream to suppress their expression, tumors with higher baseline expression (e.g., MSI or CIN subtypes) might show greater sensitivity. However, these hypotheses are derived solely from public database analyses and require prospective clinical validation.

We subsequently performed functional and pathway enrichment analyses on the intersected targets, revealing their close associations with biological processes such as apoptosis, proliferation, and metastasis in tumor cells. Furthermore, these targets may exert antitumor effects through the MAPK signaling pathway (44). KEGG pathway enrichment analysis has confirmed significant enrichment in the MAPK signaling pathway. Our research revealed that HL-RG is closely related to the MAPK signaling cascade, specifically the RAS/RAF/MEK/ERK signaling axis (44, 45). A recent comprehensive review highlighted that phytochemicals can modulate the MAPK pathway in gastrointestinal cancers, reducing tumor development and inducing cancer cell death (45). The novelty of our study lies in three aspects. First, unlike previous reports focusing on berberine or cinnamaldehyde alone, we investigated HL−RG as an integrated herbal pair, which better reflects clinical practice. Second, HL−RG simultaneously downregulates five hub genes (PDGFRB, EGFR, KIT, MMP2, MMP9) and suppresses the MAPK/ERK pathway, indicating a multi−target network effect. This is supported by evidence that co−administration of HL-RG enhances the bioavailability of alkaloids and cinnamic acid via P−gp inhibition (46). Third, we are the first to provide functional rescue evidence using the MEK inhibitor U0126 and the MAPK agonist EGF, demonstrating that the anti−proliferative, pro−apoptotic, and anti−metastatic effects of HL−RG depend on the MAPK/ERK signaling pathway, moving beyond correlation to causality. Thus, the innovation of our study lies in the three dimensions of herbal pair integration, multi−target synergy, and causal validation.

After HL - RG intervention in gastric cancer cells, the expression of RAS, ERK1, and ERK2 was reduced at the mRNA level, and upstream factors that activate the MAPK pathway, such as EGFR, PDGFRB, and KIT, as well as the downstream factor MMP2, were also decreased. Moreover, Western blot experiments confirmed that the expression of RAS and p-ERK also decreased at the protein level. Our experimental results confirm that HL-RG can inhibit the proliferation and metastasis of GC cells by regulating the MAPK signaling pathway, More importantly, to establish causality, we performed rescue experiments using the MEK inhibitor U0126 and the MAPK agonist EGF. The finding that U0126 alone phenocopied HL−RG effects, while EGF significantly reversed HL−RG−induced functional changes and p−ERK reduction, provides direct functional evidence that HL−RG acts through the MAPK/ERK pathway rather than through off−target or multi−pathway mechanisms.

In addition, we observed that 5−FU, a standard chemotherapeutic agent, caused significantly greater damage to normal gastric mucosal GES−1 cells than HL−RG in the CCK−8 assay. While 5−FU inhibits DNA synthesis and effectively suppresses GC cell growth, it often inadvertently harms normal cells. In contrast, HL−RG not only inhibited proliferation and migration of GC cells but also showed lower toxicity to normal gastric epithelial cells, suggesting a favorable safety profile.

In summary, through network pharmacological and bioinformatics analyses, we confirmed that the combination of HL-RG exerts antitumor effects on GC by acting on the MAPK signaling pathway. This combination inhibits GC cell proliferation, induces apoptosis, suppresses angiogenesis (40, 45).

However, our research has several limitations. First, Although our rescue experiments using U0126 and EGF strongly support MAPK dependency, we did not perform siRNA−mediated knockdown of individual hub genes (EGFR or MMP9). Future studies will employ genetic approaches to further dissect the specific contribution of each hub gene to HL−RG action. Second, all functional experiments in our study were conducted *in vitro* using GC cell lines, and no *in vivo* studies have been conducted to date. In future experiments, we plan to establish a nude mouse xenograft model to evaluate the antitumor efficacy and systemic toxicity of HL-RG (47). Third, the molecular docking predictions in this study are purely computational. Although we performed redocking validation (RMSD < 2.0 Å) to confirm the reliability of our docking parameters, direct biophysical assays (e.g., surface plasmon resonance, SPR, or microscale thermophoresis, MST) are required to confirm the physical binding between HL−RG components (e.g., berberine) and hub proteins (e.g., EGFR, MMP2). We plan to conduct such experiments in future studies. Co-IP assays, ChIP assays, or gene silencing methods (lentiviral transfection techniques) should be used to validate the causal relationships among upstream and downstream proteins within the MAPK signaling pathway (48). Combined with dual-luciferase and chromatin immunoprecipitation assays, the regulatory effect of ERK on GC should be further investigated to fully elucidate the specific antitumor mechanism of HL-RG (49).

## Data Availability

Publicly available datasets were analyzed in this study. This data can be found here: GEO dataset GSE65801 (https://www.ncbi.nlm.nih.gov/geo/query/acc.cgi?acc=GSE65801); GeneCards (https://www.genecards.org); DisGeNET (https://www.disgenet.org).
