# Supplementary material for: Exploring the molecular mechanism of Coptis-cinnamon in combating gastric cancer via the MAPK Pathway based on network pharmacology
Source: Front Oncol. 2026 May 14;16:1824009. doi: 10.3389/fonc.2026.1824009 (PMC13217296; doi:10.3389/fonc.2026.1824009)
Supplement: Supplementary file 1 [file DataSheet1.docx]

**Supplementary Materials**

**Supplementary Table 1.** Active compounds information.

| **Mol ID** | **Molecule Name** | **OB(%)** | **DL** | **SMILES** |
| --- | --- | --- | --- | --- |
| MOL001454 | berberine | 36.86 | 0.78 | COC1=C(C2=C[N+]3=C(C=C2C=C1)C4=CC5=C(C=C4CC3)OCO5)OC |
| MOL002894 | berberrubine | 35.74 | 0.73 | COC1=C(C2=C[N+]3=C(C=C2C=C1)C4=CC5=C(C=C4CC3)OCO5)O |
| MOL002664 | Fagarine | 72.23 | 0.15 | COC1=CC=CC2=C1N=C3C(=C2OC)C=CO3 |
| MOL013352 | Obacunone | 43.29 | 0.77 | CC1(C2CC(=O)C3(C(C2(C=CC(=O)O1)C)CCC4(C35C(O5)C(=O)OC4C6=COC=C6)C)C)C |
| MOL002897 | epiberberine | 43.09 | 0.78 | COC1=C(C=C2C(=C1)CC[N+]3=C2C=C4C=CC5=C(C4=C3)OCO5)OC |
| MOL002903 | (R)-Canadine | 55.37 | 0.77 | COC1=C(C2=C(CC3C4=CC5=C(C=C4CCN3C2)OCO5)C=C1)OC |
| MOL002904 | Berlambine | 36.68 | 0.82 | COC1=C(C2=C(C=C1)C=C3C4=CC5=C(C=C4CCN3C2=O)OCO5)OC |
| MOL002907 | Corchoroside A_qt | 104.95 | 0.78 | C[C@]12CC[C@H]3[C@@H](CC[C@]4(O)C[C@H](O)CC[C@@]34C=O)[C@@]1(O)CC[C@H]2C1=CC(=O)OC1 |
| MOL000622 | Magnograndiolide | 63.71 | 0.19 | CC1(CCC2C(C3C1CCC3(C)O)OC(=O)C2=C)O |
| MOL000785 | palmatine | 64.6 | 0.65 | COC1=C(C2=C[N+]3=C(C=C2C=C1)C4=CC(=C(C=C4CC3)OC)OC)OC |
| MOL000098 | quercetin | 46.43 | 0.28 | C1=CC(=C(C=C1C2=C(C(=O)C3=C(C=C(C=C3O2)O)O)O)O)O |
| MOL001458 | coptisine | 30.67 | 0.86 | C1C[N+]2=C(C=C3C=CC4=C(C3=C2)OCO4)C5=CC6=C(C=C51)OCO6 |
| MOL002668 | Worenine | 45.83 | 0.87 | CC1=C2C3=CC4=C(C=C3CC[N+]2=CC5=CC6=C(C=C15)OCO6)OCO4 |
| MOL008647 | Moupinamide | 86.71 | 0.26 | COC1=C(C=CC(=C1)C=CC(=O)NCCC2=CC=C(C=C2)O)O |
| MOL002003 | (-)-Caryophyllene oxide | 32.67 | 0.13 | CC1(CC2C1CCC3(C(O3)CCC2=C)C)C |
| MOL000057 | DIBP | 49.63 | 0.13 | CC(C)COC(=O)C1=CC=CC=C1C(=O)OCC(C)C |

**Supplementary Figure 1.** Hub genes are involved in epigenetic regulation and repair of damaged genes.

A


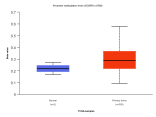

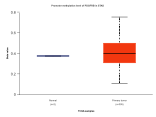

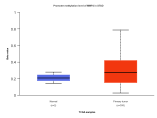

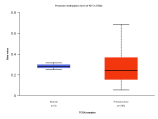

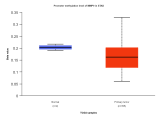

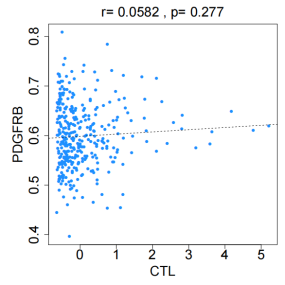

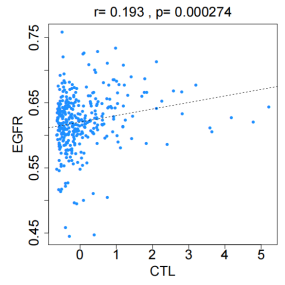

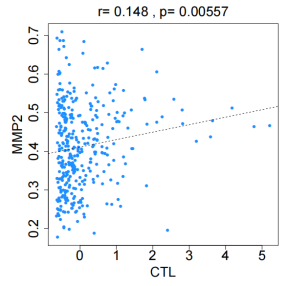

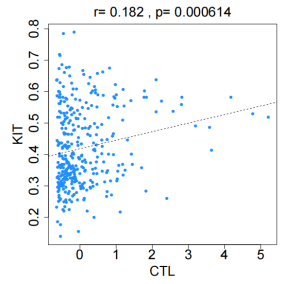

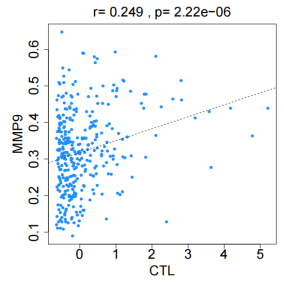


B

C


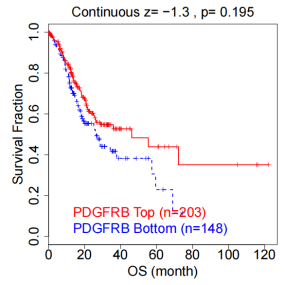

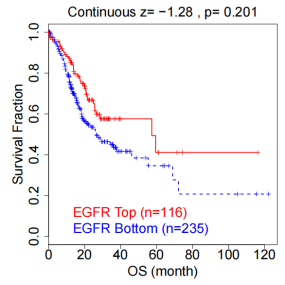

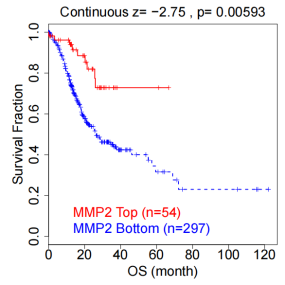

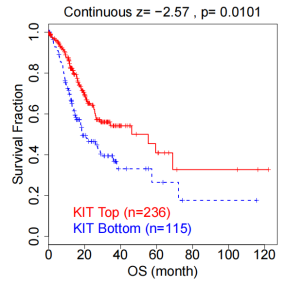

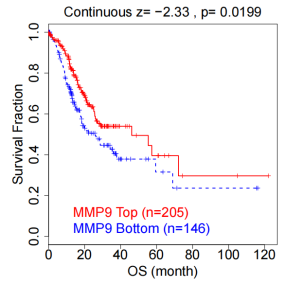


D

E


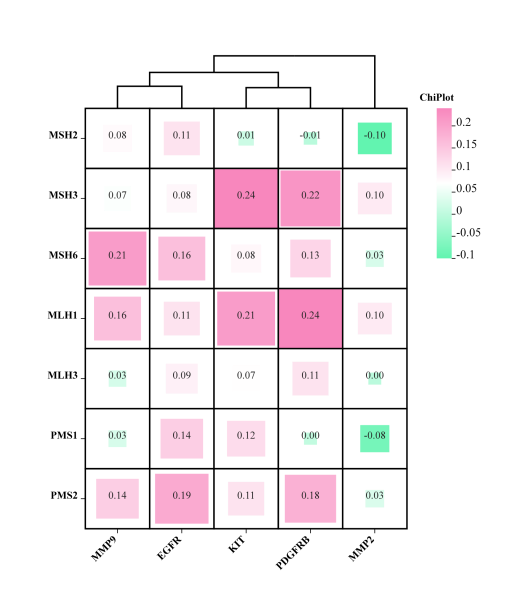

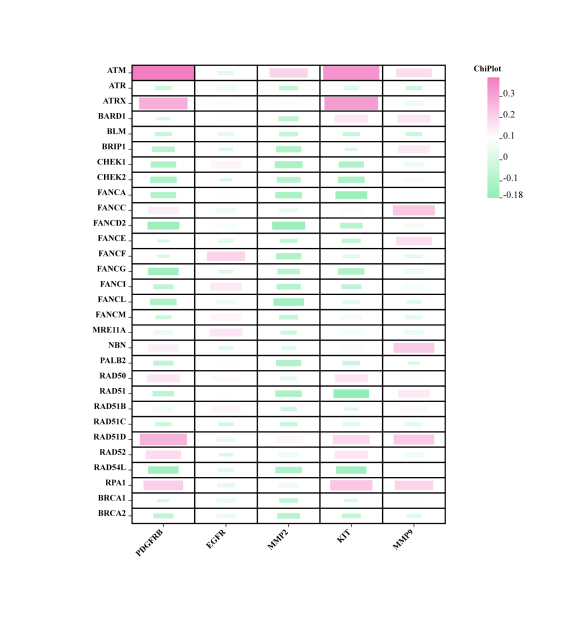


(**A**) Expression level map of Hub gene methylation. Blue and red represent the normal and tumor groups, respectively.

(**B**) Correlation between Hub gene methylation levels and CTL markers.

(**C**) Survival curves of hypermethylated and hypomethylated subgroups of Hub genes were plotted.

(**D-E**) Heatmap of correlation between Hub genes and HRR and MMR repair systems. Pink color represents positive correlation and green color represents negative correlation. The size of the box represents the magnitude of the correlation; the larger the box, the stronger the correlation.

**Supplementary Figure 2.** Relationship between Hub genes and immune infiltration.

A


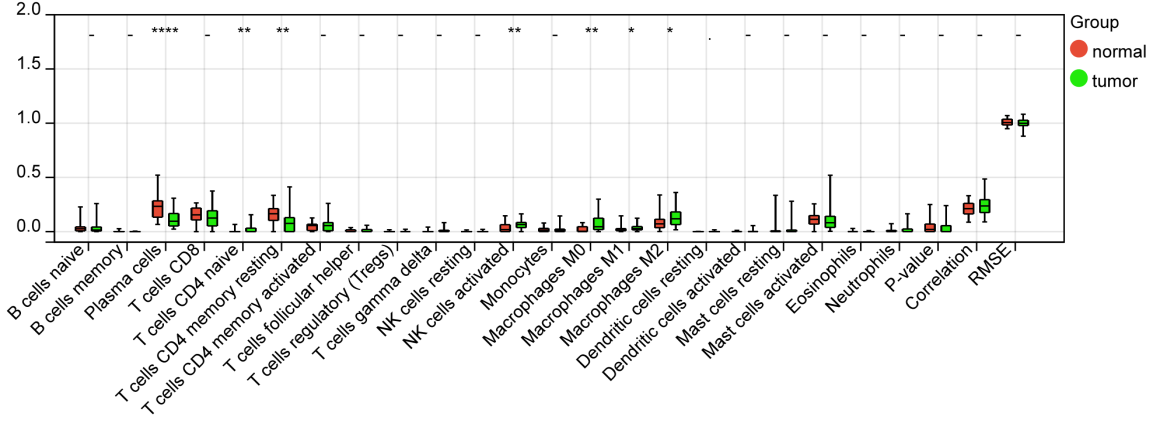


C


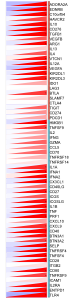


PDGFRB EGFR MMP2 KIT MMP9


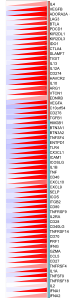

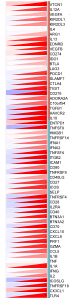

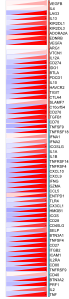

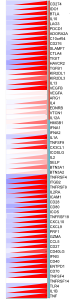

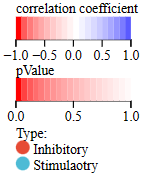

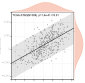

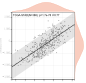

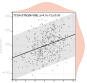

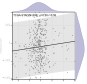

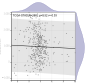

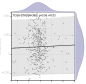

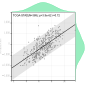

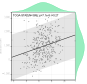

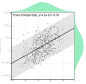

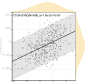

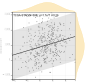

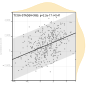

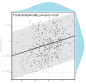

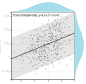

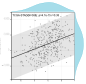


PDGFRB

EGFR

MMP2

KIT

MMP9

StromalScore Immunescore ESTIMATEScore

B


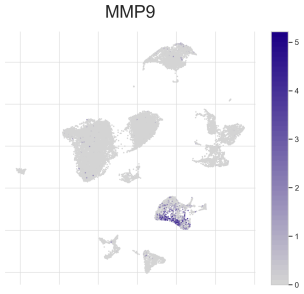

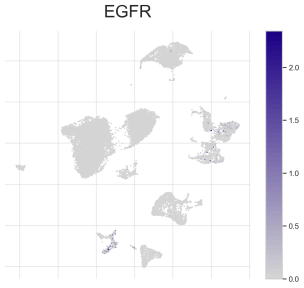

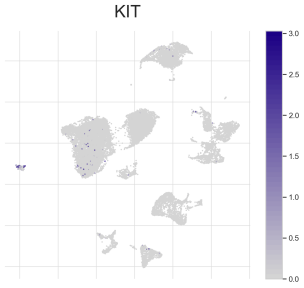

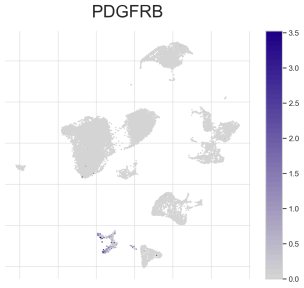

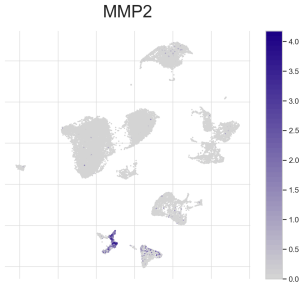

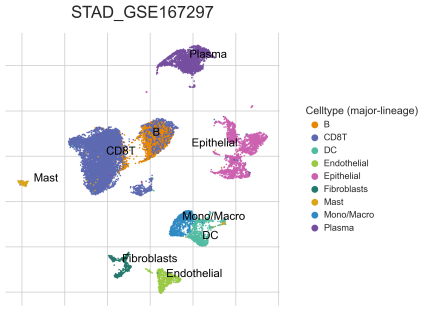


D

E


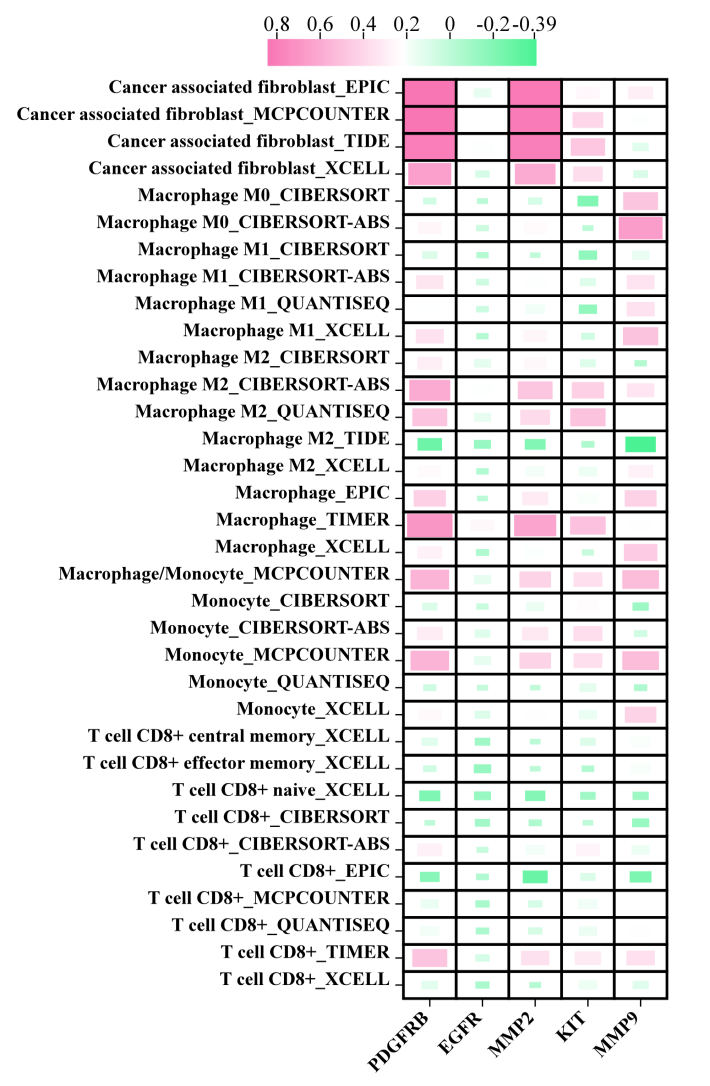


(**A**)Immune cell infiltration in gastric cancer was calculated using CIBERSORT. **P < 0.05,*  ***P < 0.01,* ****P < 0.001,* *****P < 0.0001*.

(**B**)Scatter plot of StromalScore, ImmuneScore, ESTIMATEScore correlation of Hub gene

(**C**)Heatmap of correlation between Hub genes and immune checkpoints. Blue color represents negative correlation and red color represents positive correlation.

(**D**)Single-cell sequencing (top) and single-cell annotation map (bottom) of Hub genes. Different colors represent different sets of cells.

(**E**)Hub gene correlates with macrophage, endothelial cell, tumor-associated fibroblast, and CD8+ T cell infiltration. Green represents negative correlation, red represents positive correlation, and larger bubbles and darker colors represent stronger correlation.
